# Effectiveness of an Internet-Based Self-Guided Program to Treat Depression in a Sample of Brazilian Users: Randomized Controlled Trial

**DOI:** 10.2196/46326

**Published:** 2023-08-17

**Authors:** Rodrigo T Lopes, Gustavo Chapetta da Rocha, Maria Adriana Svacina, Björn Meyer, Dajana Šipka, Thomas Berger

**Affiliations:** 1 University of Bern Bern Switzerland; 2 Catholic University of Petropolis Petrópolis Brazil; 3 Gaia Group Hamburg Germany

**Keywords:** depression, internet-based interventions, self-guided interventions, cognitive behavioral therapy

## Abstract

**Background:**

Depression is undertreated in Brazil. Deprexis is a self-guided internet-based program used to treat depressive symptoms based on empirically supported integrative and cognitive behavioral therapy. Evidence from a meta-analysis supports Deprexis’ efficacy in German-speaking countries and the United States, but no study has been conducted using this program in countries with low literacy rates and large social disparities. Furthermore, few studies have investigated whether internet-based interventions ameliorate the psychological processes that might underlie depressive symptomatology, such as low perceived self-efficacy.

**Objective:**

The main objective of this study was to replicate in Brazil previously reported effects of Deprexis on depressive symptom reduction. Therefore, the main research question was whether Deprexis is effective in reducing depressive symptoms and the general psychological state in Brazilian users with moderate and severe depression in comparison with a control group that does not receive access to Deprexis. A secondary research question was whether the use of Deprexis affects perceptions of self-efficacy.

**Methods:**

We interviewed 312 participants recruited over the internet and randomized 189 participants with moderate to severe depression (according to the Patient Health Questionnaire–9 and a semistructured interview) to an intervention condition (treatment as usual plus immediate access to Deprexis for 90 days, n=94) or to a control condition (treatment as usual and delayed access to Deprexis, after 8 weeks, n*=*95).

**Results:**

Participants from the immediate access group logged in at Deprexis an average of 14.81 (SD 12.16) times. The intention-to-treat analysis using a linear mixed model showed that participants who received Deprexis improved significantly more than participants assigned to the delayed access control group on the primary depression self-assessment measure (Patient Health Questionnaire–9; Cohen *d*=0.80; *P*<.001) and secondary outcomes, such as general psychological state measure (Clinical Outcome in Routine Evaluation–Outcome Measurement; Cohen *d*=0.82; *P*<.001) and the perceived self-efficacy measure (Cohen *d*=0.63; *P*<.001). The intention-to-treat analyses showed that 21% (20/94) of the participants achieved remission compared with 7% (7/95) in the control group (*P*<.001). The deterioration rates were lower in the immediate access control group. The dropout rate was high, but no differences in demographic and clinical variables were found. Participants reported a medium to high level of satisfaction with Deprexis.

**Conclusions:**

These results replicate previous findings by showing that Deprexis can facilitate symptomatic improvement over 3 months in depressed samples of Brazilian users. From a public health perspective, this is important information to expand the reach of internet-based interventions for those who really need them, especially in countries with less access to mental health care. This extends previous research by showing significant effects on perceived self-efficacy.

**Trial Registration:**

Registro Brasileiro de Ensaios Clíncos (ReBec) RBR-6kk3bx UTN U1111-1212-8998; https://ensaiosclinicos.gov.br/rg/RBR-6kk3bx/

**International Registered Report Identifier (IRRID):**

RR2-10.1590/1516-4446-2019-0582

## Introduction

### Background

Depression is a main concern for public health and one of the main causes of functional impairment and is associated with immense personal distress and increased suicide risk. Although depressive disorders are among the most common mental health problems for which help is sought [1,2], there is evidence suggesting that those who most need help (eg, those with more severe depressive symptoms) are those who least seek treatment [3]. General barriers to providing psychotherapy include high costs for clients, long commutes, time constraints, negative perceptions of psychotherapy, and limited availability of services [4,5]. Importantly, in many parts of the world, there is a shortage of qualified professionals, especially in rural or sparsely populated areas [6]. Even in urban centers, affordable services often have long waitlists. Many clients also do not seek help because of the stigma associated with psychological and psychopharmacological treatments [7]. Depression-related barriers have also been described, such as a lack of motivation, emotional concerns [4], and negative perceptions of psychotherapy effectiveness [3]. In addition, treatment coverage varies according to wealth distribution in the world. The current treatment coverage for upper middle–income countries, such as Brazil, is estimated to be only 21% of people with depression, which results in a treatment gap of 79% of people with untreated depression [8]. To overcome these access barriers and improve the quality of depression care, treatments delivered over the internet have gained popularity over the last 2 decades [9].

Research suggests that internet-based interventions could help address the treatment gap because, when compared with traditional face-to-face formats, (1) they can be more cost-effective [10]; (2) they are accessible at a lower threshold [11]; (3) they can be used more flexibly, independent of time and place [11]; (4) they are more anonymous and private, which is attractive for individuals with fear of stigmatization [12]; and (5) they are easily translatable and adaptable to other cultures [13]. In Brazil, research on such interventions is still in its early stages and very few studies have assessed the effectiveness of internet-based treatments. We are not aware of any studies in Brazil that have evaluated the effectiveness of an internet-based intervention for psychological problems in a controlled trial. Thus, more studies on the results and processes of internet-based treatments are necessary to better evaluate their impact on potential users in the Brazilian context [14].

Deprexis is an established, self-guided internet-based intervention for adults with elevated depressive symptoms. It uses an integrative approach based primarily on cognitive behavioral therapy (CBT) [15]. A recent Deprexis-specific meta-analysis of 12 randomized controlled trials reported an average effect size of 0.51 (Hedges *g*) after 8 to 12 weeks, and the effectiveness of this program was not significantly associated with the level of clinician guidance, developer involvement, setting (community vs clinical), and initial symptom severity [16]. Additional studies have also demonstrated that intervention effects generalize to routine care settings [17] and that the intervention is cost-effective [10]. Evidence for the efficacy of this program has been reported in German-speaking countries [3,10,15,18-26]) as well as in the United States [27]. All previous studies were conducted in high-income countries (according to the World Bank) [28]. Although Deprexis has been translated into 10 languages, including Brazilian Portuguese, no study has been conducted with Brazilians who have depression.

Although much research has shown the impact of internet-based interventions on depression reduction, less is known about the effects of such programs on potentially underlying psychological variables such as perceptions of self-efficacy. General self-efficacy is the perception that one has about one’s general ability to carry out the actions required to reach important goals, overcome obstacles or difficulties, and succeed [29,30]. Certain self-efficacy perceptions may play key roles in the pathogenesis and recurrence of depressive episodes, including (1) perceptions of being incapable of achieving satisfying standards of performance, (2) perceptions of being unable to cultivate or maintain satisfying interpersonal relationships, and (3) perceptions of being unable to regulate or control depressive ruminations [31]. Therefore, it would be of theoretical and practical interest to determine whether digital treatment programs not only reduce depressive symptoms but also improve potentially underlying cognitive processes, such as low self-efficacy perceptions. Some studies suggest that psychological treatments may indeed improve self-efficacy perceptions, which might mediate treatment effects on symptomatic improvement [32], but the findings appear to be mixed [33]. There is also evidence from a qualitative study (n*=*14) that using a CBT program for depression at a self-determined pace helped participants gain control of their lives and feel more competent and autonomous [34], which suggests improvements in self-efficacy perceptions. However, no previous studies have examined whether a self-guided digital intervention, such as Deprexis, can enhance self-efficacy perceptions among Brazilian users with elevated depression. Therefore, we aimed to explore the connections between the participants’ self-efficacy perceptions and their depression levels before and after the intervention.

### Objectives

The main objective of this study was to replicate in Brazil previously reported effects of Deprexis on depressive symptom reduction. Therefore, the main research question was whether Deprexis is effective in reducing depressive symptoms and general psychological state in Brazilian users with moderate and severe depression in comparison with a control group that does not receive access to Deprexis. A secondary research question was whether the use of Deprexis affects the perceptions of self-efficacy.

## Methods

### Design

We used a randomized controlled trial parallel-group design (trial registration at Registro Brasileiro de Ensaios Clíncos [ReBec]: RBR-6kk3bx, UTN U1111-1212-8998) [35]. Participants were randomly allocated into two conditions: (1) an experimental condition in which they received treatment as usual (TAU) plus immediate access to Deprexis for 90 days or (2) a control group, in which they also received TAU and delayed access to Deprexis after 8 weeks. The participants were not blinded to the condition in which they were randomized. TAU (other psychological or psychopharmacological treatments) was assessed before and after treatment.

### Recruitment

A website, a fan page on Facebook, an Instagram account, and a Twitter profile (all IDs: @p.ficabem) were set up to provide basic information about the research procedures, about the internet-based intervention, and to recruit participants. The link to the website was advertised through associations with mental health professionals in Brazil (via email or WhatsApp). In addition, members of the research team were interviewed on local and regional television channels and radio stations. The recruitment of participants was conducted between August 2018 (first participant in) and September 2020 (last participant in). From around halfway through the period (October 2019), we sponsored the link on the website using Google Ads. With this tool, the links to our study appeared in YouTube videos of mental health content.

### Inclusion and Exclusion Criteria

Eligible participants (1) were at least 18 years of age; (2) had regular access to the internet; (3) were residents of Brazil; (4) presented clinically relevant depressive symptoms defined as a score of at least 10 on the Patient Health Questionnaire–9 (PHQ-9) [36,37]; and (5) were diagnosed with a depressive disorder or dysthymia following the definitions of Diagnostic and Statistical Manual of Mental Disorders, Fifth Edition (DSM-5) [38]. Participants were allowed to be in other psychological or psychopharmacological TAU if treatments were stabilized (ie, treatments lasted at least 1 month). Candidates were excluded from the study if the screening interview indicated that they (1) presented other severe psychiatric symptoms that should be the primary focus of clinical attention (ie, severe psychotic symptoms, manic episodes, severe substance abuse, and severe obsessive compulsive disorder), (2) showed the potential to harm themselves or others, (3) had severe suicidal ideation, and (4) were psychiatric patients in the process of adaptation to medication (taking medication for less than a month, changed dosage within the past month, or intention to switch medications within 3 months after the start of the trial).

### Instruments

#### Sociodemographic Questionnaire

Demographic variables assessed were age, gender, and location in Brazil.

#### Adult DSM-5 Cross-Cutting Symptom Measure

For the initial screening, a semistructured diagnostic interview based on the diagnostic criteria of the DSM-5 was used [38]. The interviews assessed the mental health domains that are important across psychiatric diagnoses. The adult version of the measure consists of 23 questions that assess 13 psychiatric domains, including depression, anger, mania, anxiety, somatic symptoms, suicidal ideation, psychosis, sleep problems, memory, repetitive thoughts and behaviors, dissociation, personality functioning, and substance use. The original English version of the measure was found to have good-to-excellent test-retest reliability in the DSM-5 field trials conducted in adult clinical samples across the United States and Canada [39,40]. We included questions about previous diagnoses and previous and current treatments.

#### PHQ-9 Measure

The PHQ-9 served as the primary outcome measure; it is a self-report scale that assesses the presence of each of the symptoms of a major depressive episode, as described in DSM-5 [36-38]. A total score of 0 to 4 points is considered none to minimal depressive symptoms; 5 to 9 is considered mild depressive symptoms; 10 to 14 is considered moderate depressive symptoms; 15 to 19 is considered moderately severe symptoms; and 20 to 27 is considered severe depressive symptoms [36,37]. Brazilian psychiatrists translated the PHQ-9 into Portuguese, and a back translation was performed by one of the authors of the original instrument [41]. The cutoff score of 10 points is widely used by Brazilian researchers [36,42-44]. The psychometric properties of the PHQ-9 are excellent, with high internal consistency, test-retest reliability, criterion validity, comparatively favorable sensitivity and specificity, and good sensitivity to change [45]. Considering the whole sample after treatment (n=2398), the internal consistency (Cronbach α) for the PHQ-9 in this study was .82.

#### Clinical Outcome in Routine Evaluation–Outcome Measurement

The Clinical Outcome in Routine Evaluation–Outcome Measurement (CORE-OM) is a self-report scale that is widely used to evaluate the general psychological state and effectiveness of mental health treatments [46,47]. It served as a secondary outcome measure. It contains 34 items to assess 4 domains: subjective well-being (4 items), problems and symptoms (12 items), life functioning (12 items), and risk to self and others (6 items). Items are answered on a 5-point scale, ranging from “never” to “always.” A study conducted in the United Kingdom with a clinical (n=890) and a nonclinical sample (n=1106) yielded an overall index of internal consistency (Cronbach α) score of .94 for both samples [46]. Using data from the pretreatment sample that responded to the CORE-OM (n=2282), the internal consistency (Cronbach α) in this study was .91. For our analysis, we also considered the subjective well-being and life functioning scales separately.

#### General Self-Efficacy Scale

The general self-efficacy scale is a self-report measure consisting of 10 items, each scored from 1 (not at all true) to 4 (exactly true) [48,49]. Scores ranged from 10 to 40, with the highest score corresponding to the strongest self-efficacy perceptions. It served as a secondary outcome measure. Internal consistency is generally high (Cronbach α>.90) [50]. Using data from the pretreatment sample that responded to the general self-efficacy scale (n=2355), the internal consistency (Cronbach α) in this study was .90.

#### Client Satisfaction Questionnaire–8

The Client Satisfaction Questionnaire–8 (CSQ-8) is a self-report questionnaire that assesses the general level of satisfaction with the services provided [51]. It has 8 items that are scored on a scale from 1 to 4. The total score varies from 8 to 32, where a higher score indicates greater satisfaction. The internal consistency of the original English version scale ranges from 0.83 to 0.93 [52]. Using data from the sample that responded to the posttreatment questionnaires (n=76), the internal consistency (Cronbach α) for the CSQ-8 was .94.

#### Program Use

The system generated the data on the number of times the program was accessed as an objective measure of adherence to treatment.

### Procedures

All selected clients who decided to participate in the research were informed and consented to participate by clicking *yes* on the web-based informed consent form, in which they were explained the aim of the research, that Deprexis has been previously researched, that minimal risks were expected, and that they could withdraw at any time. They were then asked to complete the sociodemographic questionnaire, the PHQ-9, and the CORE-OM. To assess the exclusion and inclusion criteria, interviews based on the *Adult DSM-5 Cross-Cutting Symptom Measure* were conducted by phone, Skype, or WhatsApp (always exclusively via audio). Diagnostic interviews and minimal guidance (providing feedback about program use and motivation) were conducted by Brazilian psychology students in their senior year of bachelor’s degree (fifth year) at the Catholic University of Petropolis. They had been trained and supervised by 3 clinical psychologists who were also members of the research team. Interviewers were assigned to interviewees according to time availability. Candidates who were not eligible for the study were contacted for feedback on the initial evaluation by telephone, by email, or by the end of the interview. They were referred to public mental health face-to-face services in their residential area.

The selected participants were randomly assigned to either the immediate access condition or to the delayed access condition. A 50:50 randomization procedure was used. It was computer generated using a random algorithm developed at randomization [53]. The randomization scheme was concealed from the study team. The list was generated by the study leader before data collection, and only 1 student could open the file. Participants randomized to the immediate access group received a voucher to access the program for 90 days and were asked to complete the outcome measures after this period. They were also asked to answer a short questionnaire that included questions on whether the program had been used alone or in combination with psychotherapy or psychopharmacology. Furthermore, the participants were asked about major life events that might have occurred during the intervention period. Participants randomized to the control condition were asked, 8 weeks from the randomization date, to complete the outcome measures once more, and they received a voucher to access Deprexis.

### Treatment

Deprexis is an internet-based self-guided intervention designed to help people cope with and overcome depressive symptoms [15]. No face-to-face contact occurred during treatment. It is based on the principles of CBT and consists of 10 primary content modules plus 1 brief review module: (1) introduction to the CBT model and psychoeducation; (2) behavioral activation; (3) cognitive modification; (4) mindfulness and acceptance; (5) interpersonal and communication skills; (6) relaxation techniques, physical exercise, and lifestyle modification; (7) problem-solving methods; (8) coping with difficult childhood experiences (eg, using expressive writing and forgiveness techniques); (9) positive psychology methods; and (10) working with dreams from a cognitive behavioral perspective [15,27]. The order of module presentation and the number of modules varied somewhat (from a minimum of 6 to a maximum of 10), based on individual users’ responses within the first module. For example, users who indicated that they were not interested in discussing dreams did not receive this module, and users who reported feeling tense and desiring help with relaxation received access to the module on relaxation techniques relatively earlier. However, all users received access to the core CBT modules (ie, modules 1-6). Content is presented in a simulated dialogue format, in which users continuously choose one of several predetermined response options, and the subsequent content is automatically adapted to the needs and characteristics of the users. Moreover, Deprexis sends automatic daily messages with therapeutic content to help in everyday life (eg, becoming aware of unhelpful automatic thoughts, organizing the day, breaking down large problems into small steps, and interacting with people). Deprexis is designed for both mobile and desktop devices.

Deprexis has been carefully translated from German and adapted to the Brazilian Portuguese language and culture by expert language professionals. In addition, Brazilian psychology students and psychologists used the program and suggested language and cultural adequacies. It is possible to use the program without any contact with therapists but, similar to some previous trials [27], trained psychology students maintained minimal contact with the participants by email. The messages were short feedback on use and motivation to use the program, delivered every other week. The students kept an open channel so that the participants could report any eventual harmful effects or if they had general questions about the program. The participants were instructed to work on Deprexis each week and were informed that they would only have access to the program for 90 days.

### Sample Size

The sample size calculation in this study was based on the expected difference in the primary outcome variable (PHQ-9, which measures the severity of depressive symptoms) between the group receiving the intervention and the control group after 3 months. On the basis of an estimate of statistical power of at least 0.80 in a 2-tailed test and an α of .05, we calculated a sample size of 128 participants (64 per group) to be able to demonstrate a likely effect size of Cohen *d*=0.50 (medium effect), based on previous research with Deprexis (medium effect) [16,27]. On the basis of previous clinical trials with Deprexis [27], we anticipated a study dropout rate of 20%.

### Data Analysis

Group differences in demographic data and pretreatment measures were tested with independent 2-tailed samples *t* tests and chi-square tests, where the variables consisted of nominal data. Following the intention-to-treat (ITT) principle, data from all randomized participants were analyzed. ITT analyses were conducted using linear mixed models, which are widely used in this field of research and have been recommended because of their ability to estimate the missing data accurately [54,55]. Analyses of those who adhere to the protocol (per protocol or completer analysis) were conducted using a general linear model for repeated measures, considering the observed (actual) means and SDs. Pre-post treatment effect sizes and between-group effect sizes were evaluated using Cohen *d*.

The clinical significance of the changes observed after treatment and after the delayed period was evaluated using the method by Jacobson and Truax [56]. For participants with missing posttreatment or postdelayed period data, the last observation carried forward (LOCF) [57] method was used. Chi-square tests were used to compare the rates of clinical improvement and recovery as well as any clinically significant deterioration ratios between groups. The results were reported in accordance with the CONSORT (Consolidated Standards of Reporting Trials) guidelines [58,59].

### Ethics Approval

The research protocol was approved by the Research Ethics Committee of the Catholic University of Petropolis, which is recognized by the Brazilian Ministry of Health (CAAE # 68709517.1.0000.5281). The Code of Ethics for the Practice of Psychology in Brazil [60] and the guidelines for Internet-based Interventions of Conselho Federal de Psicologia Resolution Number 11/2018, previously mentioned [61], were also considered.

## Results

### Overview

**Figure 1:**
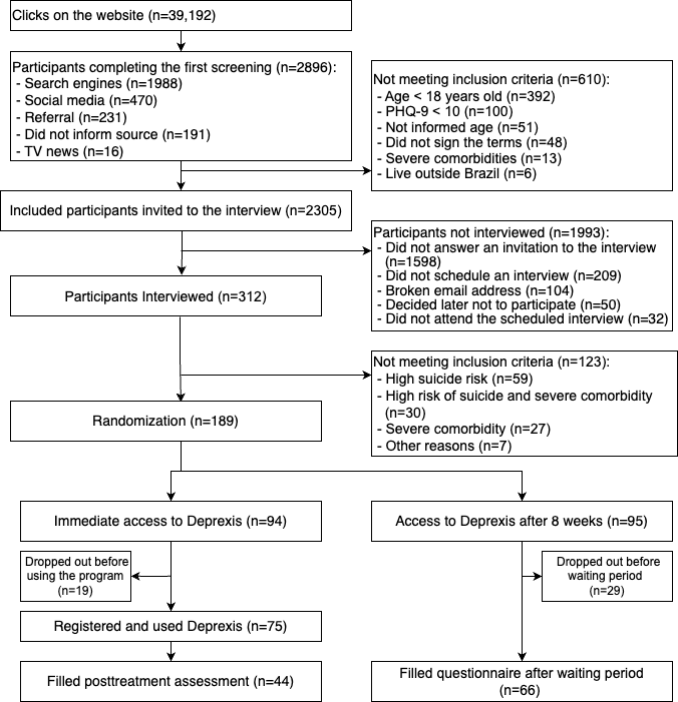
Participant flowchart. PHQ-9: Patient Health Questionnaire–9.

As shown in the flowchart (Figure 1), many interested individuals clicked on the website (n=39,192). Out of the 2896 participants who completed the first screening, 2305 were invited to participate in the interview and 312 were interviewed. Owing to the high study dropout rate (ie, participants not filling in the posttreatment assessments) and given that we had enough funding, we collected more data than the 128 initially planned [35]. We randomized 189 moderately and severely depressed participants to the immediate access condition (TAU plus immediate access to Deprexis for 90 days, n*=*94) or to the delayed access control group (TAU and delayed access to Deprexis, after 8 weeks, n*=*95).

### Sample Characteristics

The sample of 189 eligible participants consisted of 158 (83.6%) female and 31 (16.4%) male participants. The mean age of the participants was 36.44 (SD 11.52) years, ranging from 19 to 71 years. Both samples consisted mostly of participants with moderately severe and severe depression (162/189, 85.71%, considering a cutoff above 15 on the PHQ-9; mean 19.57, SD 4.81 for the immediate access group and mean 20.12, SD 4.41 for the delayed access control group; t_163_=−0.74, *P*=.74). In total, 57.7% (109/189) of the participants had been previously diagnosed with a depressive disorder. Most of them (110/189, 58.2%) did not undergo any psychological or psychiatric treatment for depression and reported other psychological disorders, mostly anxiety.

Comparing the immediate access group and the delayed access control group, there was a statistically significant difference in the participants’ recruitment method (more participants from the immediate access group came from referrals from others and more participants from the delayed access control group came from Instagram; *P*=.045). Table 1 shows the demographic and clinical characteristics of both the samples.

**Table 1:** Demographic and clinical characteristics of the sample (n=189).

Variables			Immediate access group (n=94)		Delayed access control group (n=95)		2-tailed *t* test (*df*)			Chi-square (*df*)			*P* value	
Age (years), mean (SD)			35.82 (10.76)		37.06 (12.27)		−0.09 (163)			N/A^a^			.92	
**Sex, n (%)**								N/A			0.05 (2)			.82
	Female	78 (83)		80 (84)										
	Male	16 (17)		15 (16)										
**Recruitment, n (%)**								N/A			15.8 (2)			.045
	Search engines	52 (58)		51 (60)										
	Referral	18 (20)		9 (11)										
	Facebook	9 (10)		2 (2)										
	Instagram	4 (5)		11 (13)										
	YouTube	2 (2)		4 (5)										
	WhatsApp	2 (2)		1 (1)										
	Television news	2 (2)		3 (4)										
	Twitter	0 (0)		2 (2)										
	Direct access to the website	0 (0)		2 (2)										
Completer, n (%)			44 (47)		29 (31)		N/A			9.97 (2)			.002	
Dropped out, n (%)			50 (53)		66 (69)		N/A			9.97 (2)			.002	
PHQ-9^b^ preintervention score^c^, mean (SD)			19.57 (4.81)		20.12 (4.41)		−0.74 (163)			N/A			.46	
**Severity PHQ-9, n (%)**								N/A			3.38 (2)			N/A
	Moderately severe and severe	80 (85)		82 (86)										
	Moderate	14 (15)		13 (14)										
CORE-OM^d^ preintervention score, mean (SD)			82.90 (16.34)		86.84 (17.22)		−1.74 (163)			N/A			.08	
**GSES^e^, mean (SD)**														
	Preintervention score	23.06 (6.32)		12.08 (6.94)		1.60 (163)			N/A			.11		
**Diagnosis, n (%)**								N/A			0.12 (2)			.72
	Never diagnosed	41 (44)		39 (41)										
	Diagnosed	53 (56)		56 (59)										
**Concurrent treatments, n (%)**								N/A			0.46 (2)			.50
	Not in treatment	59 (63)		54 (57)										
	Pharmacology	20 (21)		26 (27)										
	Psychotherapy	8 (9)		7 (7)										
	Both treatments	7 (8)		8 (8)										
**Comorbidities, n (%)**								N/A			0.29 (2)			.59
	No comorbidities	33 (35)		37 (39)										
	Comorbidities	61 (65)		58 (61)										
**Pandemics, n (%)**								N/A			0.00 (2)			.95
	Before	45 (48)		45 (47)										
	After	49 (52)		50 (53)										

^a^N/A: not applicable.

^b^PHQ-9: Patient Health Questionnaire–9.

^c^The PHQ-9 has a total score of 10 to 14 for moderate depressive symptoms, 15 to 19 for moderately severe depressive symptoms, and 20 to 27 for severe depressive symptoms [36,37].

^d^CORE-OM: Clinical Outcome in Routine Evaluation–Outcome Measurement.

^e^GSES: general self-efficacy scale.

### Change Over Time

The observed and estimated means for all the self-report measures are presented in Multimedia Appendix 1. Linear mixed models with the group as a fixed factor and time as a repeated factor (pre-post) were conducted separately for each outcome measure (see Multimedia Appendix 1 for the results) for the ITT sample with estimated posttreatment means. Compared with the delayed access control group, we observed significantly higher main effects for the immediate access group for the primary outcome measure (PHQ-9; *F*_1,173.5_=19.85; *P*<.001) as well as for the other secondary outcome measures (perceived general self-efficacy included; *F*_1,287_=8.278; *P*=.004).

For the completers analysis, general linear model repeated measurements showed a significant effect of time between the pre- and postassessments for the PHQ-9 (*F*_1,96_=63.14; *P*<.001) and CORE-OM scores (*F*_1_,_96_=109.11; *P*<.001), as well as a significant interaction between time and group (*F*_1,96_=16.19; *P*<.001; *F*_1,96_=8.83; *P*=.004). Symptom reduction and perceived self-efficacy improvement were significantly stronger in the immediate access group (*F*_1,216_=12.05; *P*<.001).

### Effect Sizes

Multimedia Appendix 1 also shows the pre-post within- and between-group effect sizes. For our primary outcome measure (PHQ-9), the between-group effect sizes after treatment were large, either with the estimated means (ITT analyses, Cohen *d=*0.8) or considering only the observed means (completers analysis, Cohen *d=*1.07). For the secondary outcome measures, between-group effect sizes after treatment with estimated means (ITT samples) were medium to large, ranging from Cohen *d=*0.63 (self-efficacy measure) to Cohen *d=*0.82 (CORE-OM total score). Between-group effect sizes after treatment with observed means (completers) are large, ranging from Cohen *d=*0.86 (well-being) to Cohen *d=*1.09 (general psychological state, CORE-OM total score).

### Clinical Significance Analysis

We calculated the Reliable Change Index and clinical cutoff for the PHQ-9 [56] based on our own data and compared it to other similar community samples [62]. Using the SD of 4.81 and an α of .82, the Reliable Change Index was 5.66. The cutoff score was calculated based on a general population of a Portuguese-speaking sample (criterion A=10.22) [62].

Table 2 shows that the rates of improvement of the immediate access groups were significantly higher than the control group (at *P*<.001). Considering the ITT group (n*=*189, with the pretreatment observation, carried forward to after treatment when posttreatment data were missing), 21% (20/94) attained remission and 6% (6/94) reliably improved. Within the completers group, 45% (20/44) attained remission and 14% (9/44) reliably improved. Deterioration rates were significantly higher in the delayed access control group (2 participants reliably deteriorated in the delayed access group vs none in the immediate access group).

**Table 2:** Rates of remission, improvement, overall improvement, unchanged, deterioration and reliable deterioration for intention-to-treat and treatment completers by treatment condition.

		Immediate access group, n (%)	Delayed access control group, n (%)	Chi-square (*df*)	*P* value
**Intention-to-treat group (n=189; LOCF^a^)**		n=94	n=95	29.85 (2)	<.001
	Remission^b^	20 (21)	7 (7)		
	Reliable improvement^c^	6 (6)	7 (7)		
	Minimal improvement^d^	9 (10)	28 (30)		
	Unchanged^e^	53 (56)	34 (36)		
	Minimal deterioration^f^	6 (6)	17 (18)		
	Reliable deterioration^g^	0 (0)	2 (2)		
**Treatment completers (n=110)**		n=44	n=66	22.82 (2)	<.001
	Remission^b^	20 (50)	7 (101)		
	Reliable improvement^c^	6 (14)	7 (11)		
	Minimal improvement^d^	9 (21)	28 (42)		
	Unchanged^e^	3 (7)	5 (8)		
	Minimal deterioration^f^	6 (14)	17 (26)		
	Reliable deterioration^g^	0 (0)	2 (3)		

^a^LOCF: last observation carried forward.

^b^Remission: proportion of clients who moved into the functional population (positively changed more than the Reliable Change Index=5.66 and gets under the cutoff point for PHQ-9<10).

^c^Reliable improvement: proportion of clients who positively changed more than the Reliable Change Index=5.66 and gets above the cutoff point for PHQ-9<10.

^d^Minimal improvement: proportion of clients who positively changed less than the Reliable Change Index=5.66.

^e^Unchanged: proportion of clients who maintained their PHQ-9 score.

^f^Deterioration: proportion of clients who deteriorated less than the Reliable Change Index=5.66.

^g^Reliable deterioration: proportion of clients who deteriorated more than the Reliable Change Index=5.66.

### Study Dropout

Dropout in this study was defined as not filling in the posttreatment and post–waiting period questionnaires. Completers, on the other hand, are those who filled in the posttreatment and post–waiting time questionnaires. We had 41.8% (79/189) of randomized participants dropping out of the study from before to after treatment (50/94, 53% in the immediate access group and 29/95, 31% in the delayed access control group). Of the 79 dropouts, 48 (25%) dropped out after the interview and before commencing the use of Deprexis (Figure 1). We compared the demographic and clinical characteristics of completers (n*=*110) and dropouts (n*=*79*)*. Significantly more participants randomized to the delayed access group (50/94, 53%) dropped out of the study, when compared with the ones randomized to the immediate access group (29/95, 31%; χ*^2^*_1_=9.97, *P*=.002). We found no differences with regard to age (t_177_=−1.27, *P=*.21), sex (χ*^2^*_2_=0.61, *P=*.47), recruitment source (χ*^2^*_2_=0.61, *P=*.47), intake before and after the beginning of the COVID-19 pandemic (χ*^2^*_2_=1.86, *P=*.17), having a previous mental health diagnosis (χ*^2^*_2_=1.13, *P=*.29), having comorbidities (χ*^2^*_2_=0.28, *P=*.60), being in another mental health treatment (χ*^2^*_2_=3.24, *P=*.07), baseline score of *general self-efficacy scale* (t_187_=0.24, *P=*.81), baseline score of CORE-OM (t_170_=−0.56, *P=*.58), or baseline score of PHQ-9 (t_187_=1.48, *P=*.14). Dropouts used Deprexis significantly less (mean 3.94, SD 4.14 times of use) than completers (mean 14.81, SD 12.16 times of use; t_130_=−3.60, *P*<.001).

We examined the messages exchanged by the staff and the 32 dropouts who answered the reasons for dropping out. We found that 16 participants reported that they had lost interest, 8 feared that the data would not be secure enough, 2 had problems with the log-in procedure and gave up trying, 2 reported that they no longer presented depressive symptoms, 2 stated that the questionnaires were too long, and 1 feared that he would be charged for it in the end.

### Program Use and Satisfaction

None of the participants explicitly wrote about any harmful effects of the program. The participants from the immediate access group (n*=*44) logged in to Deprexis on average 14.81 (SD 12.16) times. Participants who completed the posttreatment and postwaiting period assessment logged in to Deprexis on average 10.12 (SD 10.71) times. Participants with no posttreatment or postwaiting period logged in on average 1.54 (SD 3.21) times (t_187_=6.89, *P*<.001). The immediate access group reported a medium to high level of satisfaction with Deprexis, lying between “somewhat” (score=3) and “very satisfied” (score=4). The mean satisfaction score on the client satisfaction questionnaire was 3.4 (SD 0.64; n=44).

## Discussion

This study addressed a gap in the literature on the effectiveness of internet-based interventions for depression in countries with lower literacy rates, such as Brazil, and other linguistic and cultural backgrounds. Analyses with both the completer and the ITT samples showed that participants who received Deprexis improved significantly more than the delayed access control group on the main (depressive symptoms) and the secondary outcome measure (general psychological state), with large effect sizes. In addition, participants in the active group improved significantly more on a perceived self-efficacy measure, which yielded a medium effect size. Participants reported medium to high levels of satisfaction with Deprexis. These results replicate previous findings by showing that Deprexis can facilitate symptomatic improvement over 3 months. It also extends previous research by demonstrating that this intervention is effective in a Brazilian sample, which differs culturally and linguistically from the previously studied populations in Germany, Switzerland, and the United States.

When selecting participants, we did not establish a high-end severity cutoff; consequently, we had a severely depressed sample. This might be considered a strength of this study because few studies have been carried out with a severely depressed sample [16]. Our results showed higher effect sizes than those of other studies with participants with severe depression who had used Deprexis [18,19,24].

One novelty of this study was the finding that Deprexis facilitates changes in the perception of self-efficacy after treatment, with a medium to large effect size. This finding suggests that this treatment program not only ameliorates depressive symptom severity but may also target underlying psychological processes, such as patients’ sense of being capable of reaching important goals or mastering challenges. Self-efficacy perceptions regarding one’s capacity to perform, maintain satisfying relationships, or cope with depressive cognitions are thought to play an important role in the pathogenesis and recurrence of depressive symptoms [31], so treatments that improve such perceptions might reduce not only current depression but also the risk for future recurrences. Future studies should replicate this finding, examine whether improvements in self-efficacy mediate treatment effects, and test whether such improvements predict future symptom courses.

The LOCF method is very conservative for the ITT analysis of clinical significance. However, remission rates were 3 to approximately 4.5 times as large in the intervention when compared with the control group, depending on whether conservative LOCF analyses or completer analyses were conducted. In addition, deterioration rates were significantly higher in the delayed access control group.

We chose to recruit participants from the community over the internet, assuming that this is an important medium through which they will hear about the program under similar conditions. This introduces a bias in selecting individuals with depressive symptoms who actively seek help on the internet. However, this can also be considered the intended target group of potential Deprexis users. There was a substantial loss of people who clicked on the website to those who were sufficiently interested to complete the screening questionnaire. The reasons for this high attrition rate are unknown and should be studied in future trials.

We had a higher study dropout rate than expected, that is, 41.8% (79/189) of the randomized participants did not fill in posttreatment questionnaires. Metanalyses showed that dropout rates from internet-based treatment programs were on average 31%, ranging from 2% to 83% (Melville et al [63]). We compared the dropout and completer samples and found no differences in the demographic and clinical variables. We asked the participants about their reasons for dropping out, but their few answers did not allow us to fully comprehend this phenomenon. More studies should address the problem of dropout from internet-based interventions using creative designs. The challenges are to have clear definitions of dropping out and contacting dropout participants afterward. Given that we did not have sufficient information about the actual use of Deprexis, these results should be analyzed with caution.

For ethical reasons, the delayed access control group received Deprexis after 8 weeks of waiting time, whereas the immediate access group was evaluated at approximately 12 weeks (90 days) of Deprexis use. Although this difference might have resulted in some unknown biases, there is evidence from clinical trials that only a small proportion (12.5%) of untreated people show symptom remission after 12 weeks of the waiting period [64]. On the other hand, we consider a strength that almost half of the participants randomized to the delayed access control group were receiving other forms of TAU (psychotherapy alone, pharmacotherapy alone, or both combined). This should be a more conservative control group, in contrast to the common practice of a typical waiting list control group in which participants are selected for not having any other form of treatment. In addition, this methodological choice might improve the external validity of our results. We can assume that an individual who has depression will simultaneously seek help from different sources, either professional help or information available on the internet. In addition, Deprexis is intended to be used as an adjunct to other treatment services rather than as a replacement for existing treatments. Therefore, this add-on design is consistent with the clinical conditions under which Deprexis is offered.

Deprexis has been studied for many years and has been carefully translated and adapted to 10 different languages, including Brazilian Portuguese language and culture. These results show that this intervention is effective in different cultures even with minimal adaptation. From a public health perspective, this is important information to expand the reach of internet-based interventions for those who really need them, especially in countries with less access to mental health care. Additional research is needed to examine whether improvements are maintained over longer periods and who are particularly likely to respond to or drop out from this form of treatment.

## Appendix

Multimedia Appendix 1 Mean scores and SDs at the pretreatment, posttreatment, and postdelayed access period.

Multimedia Appendix 2 CONSORT-eHEALTH checklist.

## Abbreviations

CBT: cognitive behavioral therapy
CSQ-8: Client Satisfaction Questionnaire–8
CONSORT: Consolidated Standards of Reporting Trials
CORE-OM: Clinical Outcome in Routine Evaluation–Outcome Measurement
DSM-5: Diagnostic and Statistical Manual of Mental Disorders, Fifth Edition
ITT: intention-to-treat
LOCF: last observation carried forward
PHQ-9: Patient Health Questionnaire–9
ReBec: Registro Brasileiro de Ensaios Clíncos
TAU: treatment as usual

## Data Availability

The data set analyzed during this study is available from the corresponding author upon reasonable request.
