# Supplementary material for: Effectiveness of an Internet-Based Self-Guided Program to Treat Depression in a Sample of Brazilian Users: Randomized Controlled Trial
Source: JMIR Form Res. 2023 Aug 17;7:e46326. doi: 10.2196/46326 (PMC10472176; doi:10.2196/46326)
Supplement: Multimedia Appendix 1 [file formative_v7i1e46326_app1.docx]

## Table 2.

| **Table 2.** Mean scores and standard deviations at pre-treatment, post-treatment, and post-delayed access period. | | | | | | | | | | | | |
| --- | --- | --- | --- | --- | --- | --- | --- | --- | --- | --- | --- | --- |
|  | Pre-treatment means  (observed, completers) | | Post-treatment  (estimated) | | Post-treatment ITT between-group comparison | Post-treatment means  (observed, completers) | | Post-treatment completers between group comparison | Pre–post within-group effect sizes (estimated means) | Pre–post within-group effect sizes  (observed means, completers) | Between-group effect sizes at post-treatment (estimated means) | Between-group effect sizes at post-treatment  (observed means, completers) |
| Measure | *Mean (SD)* | *n* | *Mean (S.E.)* | *n* | *F, df and p* | *Mean (SD)* | *n* |  | Cohen’s d (95% CI) | Cohen’s d (95% CI) | Cohen’s d (95% CI) | Cohen’s d (95% CI) |
| **PHQ-9** |  |  |  |  |  |  |  |  |  |  |  |  |
| Treatment | 19.57 (4.80) | 94 | 10.56 (.95) | 94 | F _1,173.5_ = 19.85, *p*<.001 | 10.57 (6.95) | 44 | F _1,2165_ = 12.05, *p*<.001 | 1.23 (0.92-1.54) | 1.61 (1.21–2.02) | 0.80 (0.51-1.10) | 1.07 (0.67–1.48) |
| Control | 20.12 (4.41) | 95 | 17.33 (.78) | 95 |  | 17.33 (5.82) | 66 |  | 0.45 (0.16-0.74) | 0.55 (0.23–0.87) |  |  |
| **CORE-OM** |  |  |  |  |  |  |  |  |  |  |  |  |
| Treatment | 82.90 (16.34) | 87 | 51.38 (3.16) | 94 | F _3, 150.8_ = 34.07, *p*<.001 | 51.91 (24.25) | 44 | F _1,204_ = 9.87, *p*<.001 | 1.27 (0.95-1.59) | 0,97 (0.59–1.35) | 0.82 (0.53-1.12) | 1.09 (0.68–1.49) |
| Control | 86.84 (17.22) | 85 | 74.43 (2.58) | 95 |  | 75.68 (20.18) | 66 |  | 0.57 (0.27-0.87) | 0.60 (0.27–0.93) |  |  |
| **Wellbeing** |  |  |  |  |  |  |  |  |  |  |  |  |
| Treatment | 12.31 (2.54) | 86 | 7.84 (.44) | 94 | F _1,275_ = 13.12, *p*<.001 | 7.84 (3.62) | 44 | F _1,202_ = 5.68, *p*=.02 | 1.26 (0.94-1.58) | 1.48 (1.05-1.91) | 0.76 (0.47-1.06) | 0.86 (0.46-1.26) |
| Control | 12.69 (2.39) | 83 | 10.82 (.36) | 95 |  | 10.82 (3.35) | 66 |  | 0.62 (0.32-0.92) | 0.66 (0.32-0.99) |  |  |
| **Functioning** |  |  |  |  |  |  |  |  |  |  |  |  |
| Treatment | 28.47 (6.87) | 86 | 18.84 (1.15) | 94 | F _1,275_ = 10.14, *p*<.001 | 18.84 (9.71) | 44 | F _1,202_ = 7.56, *p*<.001 | 1.03 (0.72-1.34) | 1.21 (0.82-1.61) | 0.81 (0.51-1.11) | 0.99 (0.59-1.39) |
| Control | 30.71 (7.29) | 83 | 27.09 (.94) | 95 |  | 27.09 (7.30) | 66 |  | 0.43 (0.14-0.73) | 0.50 (0.17-0.82) |  |  |
| **GSES** |  |  |  |  |  |  |  |  |  |  |  |  |
| Treatment | 23.07 (6.32) | 94 | 27.70 (.97) | 94 | F _1,287_ = 8.278, *p*<.001 | 27.70 (6.20) | 44 | F _1,208_ = 5.13, *p*=.02 | 0.58 (0.29-0.87) | 0.74 (0.37-1.11) | 0.63 (0.34-0.92) | 0.92 (0.50-1.33) |
| Control | 22.08 (6.94) | 95 | 22.14 (.85) | 95 |  | 22.14 (5.97) | 58 |  | 0.01 (-0.28-0.29) | 0.01 (-0.32-0.34) |  |  |
| *Note.* ITT=Intention-to-treat; *SD*=Standard deviation; *SE=*Standard error; PHQ-9=Patient Health Questionnaire 9; CORE-OM=Clinical Outcome Routine Evaluation; GSES=General Self-efficacy scale; *CI*=Confidence Interval. Effect sizes categorized as *d* >=.2)=“small”, *d* (>=.5)=“medium”, *d* (>=.8)=“large” (Cohen, 1988). | | | | | | | | | | | | |
