# Supplementary material for: Effectiveness of an Internet-Based Self-Guided Program to Treat Depression in a Sample of Brazilian Users: Randomized Controlled Trial
Source: JMIR Form Res. 2023 Aug 17;7:e46326. doi: 10.2196/46326 (PMC10472176; doi:10.2196/46326)
Supplement: Multimedia Appendix 2 [file formative_v7i1e46326_app2.pdf]

CONSORT-EHEALTH (V 1.6.1) - Submission/Publication Form

The CONSORT-EHEALTH checklist is intended for authors of randomized trials evaluating web-based and Internet-based applications/interventions, including mobile interventions, electronic games (incl multiplayer games), social media, certain telehealth applications, and other interactive and/or networked electronic applications. Some of the items (e.g. all subitems under item 5 - description of the intervention) may also be applicable for other study designs.

The goal of the CONSORT EHEALTH checklist and guideline is to be
a) a guide for reporting for authors of RCTs,
b) to form a basis for appraisal of an ehealth trial (in terms of validity)

CONSORT-EHEALTH items/subitems are MANDATORY reporting items for studies published in the Journal of Medical Internet Research and other journals / scientific societies endorsing the checklist.

Items numbered 1., 2., 3., 4a., 4b etc are original CONSORT or CONSORT-NPT (non-pharmacologic treatment) items.
Items with Roman numerals (i., ii, iii, iv etc.) are CONSORT-EHEALTH extensions/clarifications.

As the CONSORT-EHEALTH checklist is still considered in a formative stage, we would ask that you also RATE ON A SCALE OF 1-5 how important/useful you feel each item is FOR THE PURPOSE OF THE CHECKLIST and reporting guideline (optional).

Mandatory reporting items are marked with a red *.
In the textboxes, either copy & paste the relevant sections from your manuscript into this form - please include any quotes from your manuscript in QUOTATION MARKS,
or answer directly by providing additional information not in the manuscript, or elaborating on why the item was not relevant for this study.

YOUR ANSWERS WILL BE PUBLISHED AS A SUPPLEMENTARY FILE TO YOUR PUBLICATION IN JMIR AND ARE CONSIDERED PART OF YOUR PUBLICATION (IF ACCEPTED).
Please fill in these questions diligently. Information will not be copyedited, so please use proper spelling and grammar, use correct capitalization, and avoid abbreviations.

DO NOT FORGET TO SAVE AS PDF _AND_ CLICK THE SUBMIT BUTTON SO YOUR ANSWERS ARE IN OUR DATABASE !!!

Citation Suggestion (if you append the pdf as Appendix we suggest to cite this paper in the caption):
Eysenbach G, CONSORT-EHEALTH Group
CONSORT-EHEALTH: Improving and Standardizing Evaluation Reports of Web-based and Mobile Health Interventions
J Med Internet Res 2011;13(4):e126

URL: http://www.jmir.org/2011/4/e126/
doi: 10.2196/jmir.1923
PMID: 22209829

rteixeiralopes@gmail.com Alternar conta

Não compartilhado

Rascunho salvo.

* Indica uma pergunta obrigatória

Your name *

First Last

Rodrigo T. Lopes

Primary Affiliation (short), City, Country *

University of Toronto, Toronto, Canada

University of Bern, Switzerland

Your e-mail address *

abc@gmail.com

rodrigo.lopes@unibe.ch

Title of your manuscript *

Provide the (draft) title of your manuscript.

Effectiveness of an internet-based self-guided program to treat depression in a sample of Brazilian users: results of a randomized controlled trial

Name of your App/Software/Intervention *

If there is a short and a long/alternate name, write the short name first and add the long name in brackets.

Deprexis

Evaluated Version (if any)

e.g. "V1", "Release 2017-03-01", "Version 2.0.27913"

Version 2019

Language(s) *

What language is the intervention/app in? If multiple languages are available, separate by comma (e.g. "English, French")

Portuguese

URL of your Intervention Website or App

e.g. a direct link to the mobile app on app in appstore (itunes, Google Play), or URL of the website. If the intervention is a DVD or hardware, you can also link to an Amazon page.

https://deprexis.com/

URL of an image/screenshot (optional)

https://drive.google.com/file/d/134EL32i9FtY87IzJKF88F0ZVy6adsgXx/view?usp=sharing

Accessibility *

Can an enduser access the intervention presently?

- ○ access is free and open
- ○ access only for special usergroups, not open
- ◉ access is open to everyone, but requires payment/subscription/in-app purchases
- ○ app/intervention no longer accessible
- ○ Outro:

Primary Medical Indication/Disease/Condition *

e.g. "Stress", "Diabetes", or define the target group in brackets after the condition, e.g. "Autism (Parents of children with)", "Alzheimers (Informal Caregivers of)"

Depression

Primary Outcomes measured in trial *

comma-separated list of primary outcomes reported in the trial

Depression, PHQ-9

Secondary/other outcomes

Are there any other outcomes the intervention is expected to affect?

General mental health (CORE-OM); General Self-efficacy (GSES)

Recommended "Dose" *

What do the instructions for users say on how often the app should be used?

- ○ Approximately Daily
- ◉ Approximately Weekly
- ○ Approximately Monthly
- ○ Approximately Yearly
- ○ "as needed"
- ○ Outro:

Approx. Percentage of Users (starters) still using the app as recommended after 3 months *

- ◉ unknown / not evaluated
- ○ 0-10%
- ○ 11-20%
- ○ 21-30%
- ○ 31-40%
- ○ 41-50%
- ○ 51-60%
- ○ 61-70%
- ○ 71%-80%
- ○ 81-90%
- ○ 91-100%
- ○ Outro:

Overall, was the app/intervention effective? *

- (x) yes: all primary outcomes were significantly better in intervention group vs control
- ( ) partly: SOME primary outcomes were significantly better in intervention group vs control
- ( ) no statistically significant difference between control and intervention
- ( ) potentially harmful: control was significantly better than intervention in one or more outcomes
- ( ) inconclusive: more research is needed
- ( ) Outro:

Article Preparation Status/Stage *

At which stage in your article preparation are you currently (at the time you fill in this form)

- ( ) not submitted yet - in early draft status
- ( ) not submitted yet - in late draft status, just before submission
- (x) submitted to a journal but not reviewed yet
- ( ) submitted to a journal and after receiving initial reviewer comments
- ( ) submitted to a journal and accepted, but not published yet
- ( ) published
- ( ) Outro:

Journal *

If you already know where you will submit this paper (or if it is already submitted), please provide the journal name (if it is not JMIR, provide the journal name under "other")

- ( ) not submitted yet / unclear where I will submit this
- ( ) Journal of Medical Internet Research (JMIR)
- ( ) JMIR mHealth and UHealth
- ( ) JMIR Serious Games
- ( ) JMIR Mental Health
- ( ) JMIR Public Health
- (•) JMIR Formative Research
- ( ) Other JMIR sister journal
- ( ) Outro:

Is this a full powered effectiveness trial or a pilot/feasibility trial? *

- ( ) Pilot/feasibility
- (•) Fully powered

Manuscript tracking number *

If this is a JMIR submission, please provide the manuscript tracking number under "other" (The ms tracking number can be found in the submission acknowledgement email, or when you login as author in JMIR. If the paper is already published in JMIR, then the ms tracking number is the four-digit number at the end of the DOI, to be found at the bottom of each published article in JMIR)

- ( ) no ms number (yet) / not (yet) submitted to / published in JMIR
- (•) Outro: http://doi.org/10.2196/preprints.46326

TITLE AND ABSTRACT

1a) TITLE: Identification as a randomized trial in the title

1a) Does your paper address CONSORT item 1a? *

I.e does the title contain the phrase "Randomized Controlled Trial"? (if not, explain the reason under "other")

- (•) yes
- ( ) Outro:

1a-i) Identify the mode of delivery in the title

Identify the mode of delivery. Preferably use “web-based” and/or “mobile” and/or “electronic game” in the title. Avoid ambiguous terms like “online”, “virtual”, “interactive”. Use “Internet-based” only if Intervention includes non-web-based Internet components (e.g. email), use “computer-based” or “electronic” only if offline products are used. Use “virtual” only in the context of “virtual reality” (3-D worlds). Use “online” only in the context of “online support groups”. Complement or substitute product names with broader terms for the class of products (such as “mobile” or “smart phone” instead of “iphone”), especially if the application runs on different platforms.

subitem not at all important

- 1 ( )
- 2 ( )
- 3 ( )
- 4 ( )
- 5 (•)

essential

Limpar seleção

Does your paper address subitem 1a-i? *

Copy and paste relevant sections from manuscript title (include quotes in quotation marks "like this" to indicate direct quotes from your manuscript), or elaborate on this item by providing additional information not in the ms, or briefly explain why the item is not applicable/relevant for your study

The title is "Effectiveness of an internet-based self-guided program to treat depression in a sample of Brazilian users: results of a randomized controlled trial", which addresses this topic. All the references to Deprexis (intro, Methods/Intervention) are consistent with an "internet-based self-guided program".

1a-ii) Non-web-based components or important co-interventions in title

Mention non-web-based components or important co-interventions in title, if any (e.g., “with telephone support”).

subitem not at all important

1 ○

2 ○

3 ●

4 ○

5 ○

essential

Limpar seleção

Does your paper address subitem 1a-ii?

Copy and paste relevant sections from manuscript title (include quotes in quotation marks "like this" to indicate direct quotes from your manuscript), or elaborate on this item by providing additional information not in the ms, or briefly explain why the item is not applicable/relevant for your study

There were short feedback messages to enhance participants' motivation: "It is possible to use the program without any contact with therapists but, like in some previous trials [e.g., 27], trained psychology students kept minimal contact with the participants by email. The messages were short feedbacks on usage and motivation to use the program delivered every other week."

1a-iii) Primary condition or target group in the title

Mention primary condition or target group in the title, if any (e.g., “for children with Type I Diabetes”) Example: A Web-based and Mobile Intervention with Telephone Support for Children with Type I Diabetes: Randomized Controlled Trial

subitem not at all important

1 ○

2 ○

3 ○

4 ○

5 ◉

essential

Limpar seleção

Does your paper address subitem 1a-iii? *

Copy and paste relevant sections from manuscript title (include quotes in quotation marks "like this" to indicate direct quotes from your manuscript), or elaborate on this item by providing additional information not in the ms, or briefly explain why the item is not applicable/relevant for your study

Yes, in the title it says that it is Brazilian depressed sample. We are consistent with that in the text (eg, Abstract and Methods/Participants)

1b) ABSTRACT: Structured summary of trial design, methods, results, and conclusions

NPT extension: Description of experimental treatment, comparator, care providers, centers, and blinding status.

1b-i) Key features/functionalities/components of the intervention and comparator in the METHODS section of the ABSTRACT

Mention key features/functionalities/components of the intervention and comparator in the abstract. If possible, also mention theories and principles used for designing the site. Keep in mind the needs of systematic reviewers and indexers by including important synonyms. (Note: Only report in the abstract what the main paper is reporting. If this information is missing from the main body of text, consider adding it)

subitem not at all important

1 ○

2 ○

3 ○

4 ◉

5 ○

essential

Limpar seleção

Does your paper address subitem 1b-i? *

Copy and paste relevant sections from the manuscript abstract (include quotes in quotation marks "like this" to indicate direct quotes from your manuscript), or elaborate on this item by providing additional information not in the ms, or briefly explain why the item is not applicable/relevant for your study

In the abstract: "Deprexis is a self-guided internet-based program to treat depressive symptoms based on empirically supported integrative and cognitive-behavioral therapy." Regarding the comparator, it is clearly mentioned in the abstract as well: "We randomized 189 moderately to severely depressed participants (according to the PHQ-9 and a semi-structured interview) to an intervention condition (treatment as usual [TAU] plus immediate access to Deprexis for 90 days, n=94), or to a control condition (TAU and delayed access to Deprexis, after 8 weeks, n=95)"

1b-ii) Level of human involvement in the METHODS section of the ABSTRACT

Clarify the level of human involvement in the abstract, e.g., use phrases like “fully automated” vs. “therapist/nurse/care provider/physician-assisted” (mention number and expertise of providers involved, if any). (Note: Only report in the abstract what the main paper is reporting. If this information is missing from the main body of text, consider adding it)

subitem not at all important

- 1 ○
- 2 ○
- 3 ◉
- 4 ○
- 5 ○

essential

Limpar seleção

Does your paper address subitem 1b-ii?

Copy and paste relevant sections from the manuscript abstract (include quotes in quotation marks "like this" to indicate direct quotes from your manuscript), or elaborate on this item by providing additional information not in the ms, or briefly explain why the item is not applicable/relevant for your study

The human contact was little during treatment (interview and short feedback messages to enhance motivation), as explained above

1b-iii) Open vs. closed, web-based (self-assessment) vs. face-to-face assessments in the METHODS section of the ABSTRACT

Mention how participants were recruited (online vs. offline), e.g., from an open access website or from a clinic or a closed online user group (closed usergroup trial), and clarify if this was a purely web-based trial, or there were face-to-face components (as part of the intervention or for assessment). Clearly say if outcomes were self-assessed through questionnaires (as common in web-based trials). Note: In traditional offline trials, an open trial (open-label trial) is a type of clinical trial in which both the researchers and participants know which treatment is being administered. To avoid confusion, use “blinded” or “unblinded” to indicated the level of blinding instead of “open”, as “open” in web-based trials usually refers to “open access” (i.e. participants can self-enrol). (Note: Only report in the abstract what the main paper is reporting. If this information is missing from the main body of text, consider adding it)

subitem not at all important

1 ○

2 ○

3 ○

4 ◉

5 ○

essential

Limpar seleção

Does your paper address subitem 1b-iii?

Copy and paste relevant sections from the manuscript abstract (include quotes in quotation marks "like this" to indicate direct quotes from your manuscript), or elaborate on this item by providing additional information not in the ms, or briefly explain why the item is not applicable/relevant for your study

"We randomized 189 moderately to severely depressed participants (according to the PHQ-9 and a semi-structured interview)...". We also say that they were recruited online and that PHQ-9 is self-assessed

1b-iv) RESULTS section in abstract must contain use data

Report number of participants enrolled/assessed in each group, the use/uptake of the intervention (e.g., attrition/adherence metrics, use over time, number of logins etc.), in addition to primary/secondary outcomes. (Note: Only report in the abstract what the main paper is reporting. If this information is missing from the main body of text, consider adding it)

subitem not at all important

- 1 ○
- 2 ○
- 3 ○
- 4 ◉
- 5 ○

essential

Limpar seleção

Does your paper address subitem 1b-iv?

Copy and paste relevant sections from the manuscript abstract (include quotes in quotation marks "like this" to indicate direct quotes from your manuscript), or elaborate on this item by providing additional information not in the ms, or briefly explain why the item is not applicable/relevant for your study

Participants from the immediate access group logged in at Deprexis on average 14.81 times (SD=12.16).

1b-v) CONCLUSIONS/DISCUSSION in abstract for negative trials

Conclusions/Discussions in abstract for negative trials: Discuss the primary outcome - if the trial is negative (primary outcome not changed), and the intervention was not used, discuss whether negative results are attributable to lack of uptake and discuss reasons. (Note: Only report in the abstract what the main paper is reporting. If this information is missing from the main body of text, consider adding it)

subitem not at all important

1 (selected)

2

3

4

5

essential

Limpar seleção

Does your paper address subitem 1b-v?

Copy and paste relevant sections from the manuscript abstract (include quotes in quotation marks "like this" to indicate direct quotes from your manuscript), or elaborate on this item by providing additional information not in the ms, or briefly explain why the item is not applicable/relevant for your study

The results indicate that the immediate access group had significantly higher outcomes than the delayed access access group in all measures. Eg., in the abstract "Participants from the immediate access group logged in at Deprexis on average 14.81 times (SD=12.16). Intention-to-treat analysis using a linear mixed model showed that participants who received Deprexis improved significantly more than participants assigned to the delayed access control group on the primary depression self-assessment measure (PHQ-9; Cohen's d=0.80), and secondary outcomes such as general psychological state measure (CORE-OM; d=0.82), and the perceived self-efficacy measure (d=0.63). ITT analyses show that 21.3% of the participants achieved remission against 7.4% in the control group ($p < 0.001$). Deterioration rates were lower in the immediate access control group. Dropout was high, but no differences in the demographical and clinical variables were found. Participants reported a medium to high level of satisfaction with Deprexis"

INTRODUCTION

2a) In INTRODUCTION: Scientific background and explanation of rationale

2a-i) Problem and the type of system/solution

Describe the problem and the type of system/solution that is object of the study: intended as stand-alone intervention vs. incorporated in broader health care program? Intended for a particular patient population? Goals of the intervention, e.g., being more cost-effective to other interventions, replace or complement other solutions? (Note: Details about the intervention are provided in “Methods” under 5)

subitem not at all important

1 ○

2 ○

3 ○

4 ○

5 ◉

essential

Limpar seleção

Does your paper address subitem 2a-i? *

Copy and paste relevant sections from the manuscript (include quotes in quotation marks "like this" to indicate direct quotes from your manuscript), or elaborate on this item by providing additional information not in the ms, or briefly explain why the item is not applicable/relevant for your study

"Depression is a main concern for public health and one of the main causes of functional impairment and is associated with immense personal suffering and increased suicide risk. Although depressive disorders are among the most common mental health problems for which help is sought [1,2], there is evidence suggesting that the ones who most need help (e.g., the people with more severe depressive symptoms) are the ones who least seek treatment [3]. General barriers to providing psychotherapy include high costs for clients, long commutes, time constraints, negative perceptions of psychotherapy, and limited availability of services [4,5]. Importantly, in many parts of the world, there is a shortage of qualified professionals, especially in rural or sparsely populated areas [6]. Even in urban centers, affordable services often have long wait lists. Many clients also do not seek help because of the stigma associated with psychological and psychopharmacological treatments [7]. Depression-related barriers have also been described, such as lack of motivation, emotional concerns [4], and negative perceptions of psychotherapy effectiveness [3]. Additionally, treatment coverage varies according to wealth distribution in the world. The current treatment coverage for upper-middle-income countries, such as Brazil, is estimated at only 21% of people suffering from depression, which results in a treatment gap of 79% of untreated depressed people [8]. To overcome these access barriers and improve the quality of depression care, treatments delivered over the internet have gained popularity over the last two decades [9]. Research suggests that internet-based interventions could help address the treatment gap because, when compared to traditional face-to-face formats, (1) they can be more cost-effective [10], (2) they are accessible at a lower-threshold [11], (3) they can be used more flexibly, independent of time and place [11]; (4) they are more anonymous and private, which is attractive for individuals with fear of stigmatization [12] and, also important (5) they are easily translatable and adaptable to other cultures [13]. In Brazil, research on such interventions is still at an early stage and very few studies assessing online treatments’ effectiveness have been carried out. We are not aware of any studies in Brazil that have evaluated the effectiveness of an internet-based intervention for psychological problems in a controlled trial. Thus, more studies on the results and processes of online psychotherapy treatments are necessary to better evaluate their impact on potential users in the Brazilian context [14]."

Deprexis was offered as a standalone intervention (self-guided) with minimal guidance.

2a-ii) Scientific background, rationale: What is known about the (type of) system

Scientific background, rationale: What is known about the (type of) system that is the object of the study (be sure to discuss the use of similar systems for other conditions/diagnoses, if appropiate), motivation for the study, i.e. what are the reasons for and what is the context for this specific study, from which stakeholder viewpoint is the study performed, potential impact of findings [2]. Briefly justify the choice of the comparator.

subitem not at all important

1 ○

2 ○

3 ○

4 ○

5 ◉

essential

Limpar seleção

Does your paper address subitem 2a-ii? *

Copy and paste relevant sections from the manuscript (include quotes in quotation marks "like this" to indicate direct quotes from your manuscript), or elaborate on this item by providing additional information not in the ms, or briefly explain why the item is not applicable/relevant for your study

"Deprexis is an established self-guided internet-based intervention for adults with elevated depressive symptoms. It uses an integrative approach based primarily on cognitive-behavioral therapy [CBT; 15]. A recent Deprexis-specific meta-analysis of twelve randomized controlled trials reported an average effect size of 0.51 (Hedges' g) after 8 to 12 weeks, and the effectiveness of this program was not significantly associated with level of clinician guidance, developer involvement, setting (community vs. clinical), and initial symptom severity [16]. Additional studies have also demonstrated that intervention effects generalize to routine care settings [17] and that the intervention is cost-effective [10]. Evidence for the efficacy of this program has been reported in German-speaking countries [3,10,15,18–26]) as well as in the United States [27]. All previous studies were carried out in high-income countries (according to The World Bank, n.d.). Even though Deprexis has been translated into ten languages, including Brazilian Portuguese, no study has been conducted with Brazilians who suffer from depression."

2b) In INTRODUCTION: Specific objectives or hypotheses

Does your paper address CONSORT subitem 2b? *

Copy and paste relevant sections from the manuscript (include quotes in quotation marks "like this" to indicate direct quotes from your manuscript), or elaborate on this item by providing additional information not in the ms, or briefly explain why the item is not applicable/relevant for your study

"The main objective of this study is to replicate in Brazil previously reported effects of Deprexis on depressive symptom reduction. Therefore, the main research question is whether Deprexis is effective in reducing depressive symptoms and general psychological state in Brazilian moderately and severely depressed users in comparison to a control group that does not receive access to Deprexis. A secondary research question was whether the use of Deprexis affects perceptions of self-efficacy. "

METHODS

3a) Description of trial design (such as parallel, factorial) including allocation ratio

Does your paper address CONSORT subitem 3a? *

Copy and paste relevant sections from the manuscript (include quotes in quotation marks "like this" to indicate direct quotes from your manuscript), or elaborate on this item by providing additional information not in the ms, or briefly explain why the item is not applicable/relevant for your study

Yes. "We used a randomized controlled trial (RCT) parallel-group design (trial registration at ReBec: RBR-6kk3bx, UTN U1111-1212-8998) [34]. Participants were randomly allocated into two conditions: (1) an experimental condition, in which they received treatment as usual (TAU) plus immediate access to Deprexis for 90 days or (2) a control group, in which they also received TAU and delayed access to Deprexis after 8 weeks. TAU (other psychological or psychopharmacological treatments) was assessed at pre- and post-treatment."

3b) Important changes to methods after trial commencement (such as eligibility criteria), with reasons

Does your paper address CONSORT subitem 3b? *

Copy and paste relevant sections from the manuscript (include quotes in quotation marks "like this" to indicate direct quotes from your manuscript), or elaborate on this item by providing additional information not in the ms, or briefly explain why the item is not applicable/relevant for your study

NA. The trial is consistent with the trial registration https://ensaiosclinicos.gov.br/rg/RBR-6kk3bx/

3b-i) Bug fixes, Downtimes, Content Changes

Bug fixes, Downtimes, Content Changes: ehealth systems are often dynamic systems. A description of changes to methods therefore also includes important changes made on the intervention or comparator during the trial (e.g., major bug fixes or changes in the functionality or content) (5-iii) and other “unexpected events” that may have influenced study design such as staff changes, system failures/downtimes, etc. [2].

subitem not at all important

- 1 (selected)
- 2
- 3
- 4
- 5

essential

Limpar seleção

Does your paper address subitem 3b-i?

Copy and paste relevant sections from the manuscript (include quotes in quotation marks "like this" to indicate direct quotes from your manuscript), or elaborate on this item by providing additional information not in the ms, or briefly explain why the item is not applicable/relevant for your study

Deprexis is an established intervention and we are not aware of changes made during the trial

4a) Eligibility criteria for participants

Does your paper address CONSORT subitem 4a? *

Copy and paste relevant sections from the manuscript (include quotes in quotation marks "like this" to indicate direct quotes from your manuscript), or elaborate on this item by providing additional information not in the ms, or briefly explain why the item is not applicable/relevant for your study

Yes. "Inclusion and exclusion criteriaEligible participants (1) were at least 18 years of age; (2) had regular access to the internet; (3) were residents of Brazil; (4) presented clinically relevant depressive symptoms defined as a score of at least 10 on the Patient Health Questionnaire-9 [PHQ-9; 35,36] and (5) were diagnosed with a depressive disorder (MDD) and/or dysthymia following the definitions of DSM 5 [37]. Participants were allowed to be in other psychological or psychopharmacological TAU if treatments were stabilized (i.e., treatments lasted at least one month). Candidates were excluded from the study if the screening interview indicated that they (1) presented other severe psychiatric symptoms that should be the primary focus of clinical attention (i.e., severe psychotic symptoms, manic episodes, severe substance abuse, severe obsessive-compulsive disorder); (2) showed potential to harm themselves or others; (3) had severe suicidal ideation; and (4) were psychiatric patients in the process of adaptation to medication (taking medication for less than a month, or changed dosage within the past month, or intending to switch medications within the three months after the start of the trial)."

4a-i) Computer / Internet literacy

Computer / Internet literacy is often an implicit “de facto” eligibility criterion - this should be explicitly clarified.

subitem not at all important

1 (selected)

2

3

4

5

essential

Limpar seleção

Does your paper address subitem 4a-i?

Copy and paste relevant sections from the manuscript (include quotes in quotation marks "like this" to indicate direct quotes from your manuscript), or elaborate on this item by providing additional information not in the ms, or briefly explain why the item is not applicable/relevant for your study

We did not access specifically computer/internet literacy, but rather we selected participants only if they "had regular access to the internet" (p. 4, Methods).

4a-ii) Open vs. closed, web-based vs. face-to-face assessments:

Open vs. closed, web-based vs. face-to-face assessments: Mention how participants were recruited (online vs. offline), e.g., from an open access website or from a clinic, and clarify if this was a purely web-based trial, or there were face-to-face components (as part of the intervention or for assessment), i.e., to what degree got the study team to know the participant. In online-only trials, clarify if participants were quasi-anonymous and whether having multiple identities was possible or whether technical or logistical measures (e.g., cookies, email confirmation, phone calls) were used to detect/prevent these.

subitem not at all important

- 1 ○
- 2 ○
- 3 ○
- 4 ◉
- 5 ○

essential

Limpar seleção

Does your paper address subitem 4a-ii? *

Copy and paste relevant sections from the manuscript (include quotes in quotation marks "like this" to indicate direct quotes from your manuscript), or elaborate on this item by providing additional information not in the ms, or briefly explain why the item is not applicable/relevant for your study

Recruitment was only online and is described in the Methods, p. 4. "A website, a fan page on Facebook, an Instagram account, and a Twitter profile (all @p.ficabem) were set up to provide basic information about the research procedures, the online intervention, and to recruit participants. The link to the website was advertised through associations of mental health professionals in Brazil (via e-mail or WhatsApp). Also, members of the research team were interviewed on local and regional TV channels and radio stations. Recruitment of participants took place between August 2018 (first participant in) and September 2020 (last participant in). From around halfway through the period (October 2019), we sponsored the link of the website using Google Ads. With this tool, the links to our study appeared in YouTube videos of mental health content"It is mentioned several times that the treatment was through the internet and it was self-guided, eg, p. 6, Treatment section: "Deprexis is an internet-based self-guided intervention designed to help people cope with and overcome depressive symptoms [15]. No face-to-face contact took place during treatment."

4a-iii) Information giving during recruitment

Information given during recruitment. Specify how participants were briefed for recruitment and in the informed consent procedures (e.g., publish the informed consent documentation as appendix, see also item X26), as this information may have an effect on user self-selection, user expectation and may also bias results.

subitem not at all important

1 ○

2 ○

3 ◉

4 ○

5 ○

essential

Limpar seleção

Does your paper address subitem 4a-iii?

Copy and paste relevant sections from the manuscript (include quotes in quotation marks "like this" to indicate direct quotes from your manuscript), or elaborate on this item by providing additional information not in the ms, or briefly explain why the item is not applicable/relevant for your study

The recruitment sources "website, a fan page on Facebook, an Instagram account, and a Twitter profile (all @p.ficabem)" was only set to "provide basic information about the research procedures and the online intervention". We told them we are as a team, that it was free of charge, the host institution, some info about depression and very shortly about the Deprexis (including a short video). This can all be seen here https://sites.google.com/view/ficabem/o-projeto?authuser=4In the paper we say that "All selected clients who decide to be part of the research were informed and consented to participate by signing an Informed Consent Form, in which they were explained the aim of the research, that Deprexis has been previously researched, that minimal risks were expected and they could withdraw at any time." (p. 8, Ethics). The informed consent form was as follows: "You are being invited to participate in the research project "Effectiveness of a self-guided, internet-based program for treating depression in a sample of Brazilian users", under the responsibility of researchers Dr. Rodrigo Teixeira Lopes (Catholic University of Petrópolis), Dr. Björn Meyer (Gaia Group, City University of London) and Dr. Américo de Araujo Pastor Junior (Federal University Fluminense). This study is important to expand and make cheaper and more accessible options for psychological treatment of depression, which is considered a serious public health problem. Deprexis has already proven effective for most people facing problems similar to yours who have used the program minimally through rigorous research conducted in Germany and the United States. If you agree to participate in this research, our quid pro quo is your free and unrestricted use of the Deprexis platform for 90 days. Your contribution is to do the proposed program, fill out some forms, and be interviewed in order to tell us about your mood and your experience with this tool. You will be accompanied by psychologists and psychology students who will be available to help you. There is a minimal risk that the dialogs will cause some discomfort at some points. Overall, more than 80% of Deprexis users report that the program has helped (Meyer, 2009). About 50% of users actually improve their depressive symptoms (Meyer, 2009; Beevers, 2017). Your participation will not offer any harm to you, as the information you provide will have confidentiality preserved. Your answers may be used in the final research document, as well as in future scientific articles and publications, always in an anonymous form. Your participation is voluntary and this consent may be withdrawn at any time without prejudice to you or the continuity of the research.In case of doubts, please contact UCP by phone, (24) 2244-4000, or by e-mail at rodrigo.lopes@ucp.br.I, ____, ID nº ____________________ declare that I have been informed about the conditions of the research and agree to participate, as a volunteer, in the research project described above.Petrópolis, ____ of __________ of 20__

4b) Settings and locations where the data were collected

Does your paper address CONSORT subitem 4b? *

Copy and paste relevant sections from the manuscript (include quotes in quotation marks "like this" to indicate direct quotes from your manuscript), or elaborate on this item by providing additional information not in the ms, or briefly explain why the item is not applicable/relevant for your study

We informed participants about the host instituition on the website and the ICF, as show in the previous item.

4b-i) Report if outcomes were (self-)assessed through online questionnaires

Clearly report if outcomes were (self-)assessed through online questionnaires (as common in web-based trials) or otherwise.

subitem not at all important

1 ○

2 ○

3 ○

4 ○

5 ◉

essential

Limpar seleção

Does your paper address subitem 4b-i? *

Copy and paste relevant sections from the manuscript (include quotes in quotation marks "like this" to indicate direct quotes from your manuscript), or elaborate on this item by providing additional information not in the ms, or briefly explain why the item is not applicable/relevant for your study

In the instruments section it is made explicit which ones (including the primary and secondary outcome measures) are self-reported

4b-ii) Report how institutional affiliations are displayed

Report how institutional affiliations are displayed to potential participants [on ehealth media], as affiliations with prestigious hospitals or universities may affect volunteer rates, use, and reactions with regards to an intervention.(Not a required item – describe only if this may bias results)

subitem not at all important

1 (selected)

2

3

4

5

essential

Limpar seleção

Does your paper address subitem 4b-ii?

Copy and paste relevant sections from the manuscript (include quotes in quotation marks "like this" to indicate direct quotes from your manuscript), or elaborate on this item by providing additional information not in the ms, or briefly explain why the item is not applicable/relevant for your study

The study were carried out in a small (not prestigious) university in Brazil.

5) The interventions for each group with sufficient details to allow replication, including how and when they were actually administered

5-i) Mention names, credential, affiliations of the developers, sponsors, and owners

Mention names, credential, affiliations of the developers, sponsors, and owners [6] (if authors/evaluators are owners or developer of the software, this needs to be declared in a "Conflict of interest" section or mentioned elsewhere in the manuscript).

subitem not at all important

1 ○

2 ○

3 ○

4 ◉

5 ○

essential

Limpar seleção

Does your paper address subitem 5-i?

Copy and paste relevant sections from the manuscript (include quotes in quotation marks "like this" to indicate direct quotes from your manuscript), or elaborate on this item by providing additional information not in the ms, or briefly explain why the item is not applicable/relevant for your study

It is declared one conflict of interest "The authors declare that they have no competing interests, with the following exception: B. Meyer is employed full-time as the research director at Gaia AG, the company that developed and owns the Deprexis program. B. Meyer has not been involved in the data collection, data organization, or analysis.", p. 12.

5-ii) Describe the history/development process

Describe the history/development process of the application and previous formative evaluations (e.g., focus groups, usability testing), as these will have an impact on adoption/use rates and help with interpreting results.

subitem not at all important

1 ○

2 ○

3 ○

4 ◉

5 ○

essential

Limpar seleção

Does your paper address subitem 5-ii?

Copy and paste relevant sections from the manuscript (include quotes in quotation marks "like this" to indicate direct quotes from your manuscript), or elaborate on this item by providing additional information not in the ms, or briefly explain why the item is not applicable/relevant for your study

"Deprexis is an established self-guided internet-based intervention for adults with elevated depressive symptoms. It uses an integrative approach based primarily on cognitive-behavioral therapy [CBT; 15]. A recent Deprexis-specific meta-analysis of twelve randomized controlled trials reported an average effect size of 0.51 (Hedges' g) after 8 to 12 weeks, and the effectiveness of this program was not significantly associated with level of clinician guidance, developer involvement, setting (community vs. clinical), and initial symptom severity [16]. Additional studies have also demonstrated that intervention effects generalize to routine care settings [17] and that the intervention is cost-effective [10]. Evidence for the efficacy of this program has been reported in German-speaking countries [3,10,15,18–26]) as well as in the United States [27]. All previous studies were carried out in high-income countries (according to The World Bank, n.d.). Even though Deprexis has been translated into ten languages, including Brazilian Portuguese, no study has been conducted with Brazilians who suffer from depression"

5-iii) Revisions and updating

Revisions and updating. Clearly mention the date and/or version number of the application/intervention (and comparator, if applicable) evaluated, or describe whether the intervention underwent major changes during the evaluation process, or whether the development and/or content was “frozen” during the trial. Describe dynamic components such as news feeds or changing content which may have an impact on the replicability of the intervention (for unexpected events see item 3b).

subitem not at all important

- 1 (selected)
- 2
- 3
- 4
- 5

essential

Limpar seleção

Does your paper address subitem 5-iii?

Copy and paste relevant sections from the manuscript (include quotes in quotation marks "like this" to indicate direct quotes from your manuscript), or elaborate on this item by providing additional information not in the ms, or briefly explain why the item is not applicable/relevant for your study

The program is quite established and this is made clear while describing it. There was no update in the translated version of the program.

5-iv) Quality assurance methods

Provide information on quality assurance methods to ensure accuracy and quality of information provided [1], if applicable.

subitem not at all important

1 ○

2 ◉

3 ○

4 ○

5 ○

essential

Limpar seleção

Does your paper address subitem 5-iv?

Copy and paste relevant sections from the manuscript (include quotes in quotation marks "like this" to indicate direct quotes from your manuscript), or elaborate on this item by providing additional information not in the ms, or briefly explain why the item is not applicable/relevant for your study

The manuscript was reviewed by all authors. The results were done and reviewed by two of the authors, with supervision of a more senior author

5-v) Ensure replicability by publishing the source code, and/or providing screenshots/screen-capture video, and/or providing flowcharts of the algorithms used

Ensure replicability by publishing the source code, and/or providing screenshots/screen-capture video, and/or providing flowcharts of the algorithms used. Replicability (i.e., other researchers should in principle be able to replicate the study) is a hallmark of scientific reporting.

subitem not at all important

1 ○

2 ○

3 ◉

4 ○

5 ○

essential

Limpar seleção

Does your paper address subitem 5-v?

Copy and paste relevant sections from the manuscript (include quotes in quotation marks "like this" to indicate direct quotes from your manuscript), or elaborate on this item by providing additional information not in the ms, or briefly explain why the item is not applicable/relevant for your study

We did the analysis using the SPSS and did not save the scripts, but we are looking a way to publish the raw data

5-vi) Digital preservation

Digital preservation: Provide the URL of the application, but as the intervention is likely to change or disappear over the course of the years; also make sure the intervention is archived (Internet Archive, webcitation.org, and/or publishing the source code or screenshots/videos alongside the article). As pages behind login screens cannot be archived, consider creating demo pages which are accessible without login.

subitem not at all important

1 (selected)

2

3

4

5

essential

Limpar seleção

Does your paper address subitem 5-vi?

Copy and paste relevant sections from the manuscript (include quotes in quotation marks "like this" to indicate direct quotes from your manuscript), or elaborate on this item by providing additional information not in the ms, or briefly explain why the item is not applicable/relevant for your study

We do not own the intervention and we have left the work of maintaining it online for the developers.

5-vii) Access

Access: Describe how participants accessed the application, in what setting/context, if they had to pay (or were paid) or not, whether they had to be a member of specific group. If known, describe how participants obtained “access to the platform and Internet” [1]. To ensure access for editors/reviewers/readers, consider to provide a “backdoor” login account or demo mode for reviewers/readers to explore the application (also important for archiving purposes, see vi).

subitem not at all important

1 ○

2 ○

3 ◉

4 ○

5 ○

essential

Limpar seleção

Does your paper address subitem 5-vii? *

Copy and paste relevant sections from the manuscript (include quotes in quotation marks "like this" to indicate direct quotes from your manuscript), or elaborate on this item by providing additional information not in the ms, or briefly explain why the item is not applicable/relevant for your study

We now referred in the MS that "demos of the program can be seen online at https://de.deprexis.com/." We also mention that "Participants randomized to the immediate access group received a voucher to access the program for 90 days and were asked to fill out the outcome measures after this period. They were also asked to answer a short questionnaire that included questions on whether the program had been used alone or together with psychotherapy or psychopharmacology. Furthermore, participants were asked about major life events that might have occurred during the intervention period. Participants randomized to the control condition were asked, eight weeks from the randomization date, to fill the outcome measures once more and when done, they received a voucher to access Deprexis."

5-viii) Mode of delivery, features/functionalities/components of the intervention and comparator, and the theoretical framework

Describe mode of delivery, features/functionalities/components of the intervention and comparator, and the theoretical framework [6] used to design them (instructional strategy [1], behaviour change techniques, persuasive features, etc., see e.g., [7, 8] for terminology). This includes an in-depth description of the content (including where it is coming from and who developed it) [1],” whether [and how] it is tailored to individual circumstances and allows users to track their progress and receive feedback” [6]. This also includes a description of communication delivery channels and – if computer-mediated communication is a component – whether communication was synchronous or asynchronous [6]. It also includes information on presentation strategies [1], including page design principles, average amount of text on pages, presence of hyperlinks to other resources, etc. [1].

subitem not at all important

1 ○

2 ○

3 ◉

4 ○

5 ○

essential

Limpar seleção

Does your paper address subitem 5-viii? *

Copy and paste relevant sections from the manuscript (include quotes in quotation marks "like this" to indicate direct quotes from your manuscript), or elaborate on this item by providing additional information not in the ms, or briefly explain why the item is not applicable/relevant for your study

Delivery is described in the Treatment section. We also cite other papers of Deprexis in which the reviewers can learn more about the development.

5-ix) Describe use parameters

Describe use parameters (e.g., intended “doses” and optimal timing for use). Clarify what instructions or recommendations were given to the user, e.g., regarding timing, frequency, heaviness of use, if any, or was the intervention used ad libitum.

subitem not at all important

1 ○

2 ○

3 ○

4 ◉

5 ○

essential

Limpar seleção

Does your paper address subitem 5-ix?

Copy and paste relevant sections from the manuscript (include quotes in quotation marks "like this" to indicate direct quotes from your manuscript), or elaborate on this item by providing additional information not in the ms, or briefly explain why the item is not applicable/relevant for your study

We added in the Treatment section that "The participants were instructed to work on Deprexis each week and they were informed that they would only have access to the program for 90 days."

5-x) Clarify the level of human involvement

Clarify the level of human involvement (care providers or health professionals, also technical assistance) in the e-intervention or as co-intervention (detail number and expertise of professionals involved, if any, as well as “type of assistance offered, the timing and frequency of the support, how it is initiated, and the medium by which the assistance is delivered”. It may be necessary to distinguish between the level of human involvement required for the trial, and the level of human involvement required for a routine application outside of a RCT setting (discuss under item 21 – generalizability).

subitem not at all important

- 1 ○
- 2 ○
- 3 ○
- 4 ◉
- 5 ○

essential

Limpar seleção

Does your paper address subitem 5-x?

Copy and paste relevant sections from the manuscript (include quotes in quotation marks "like this" to indicate direct quotes from your manuscript), or elaborate on this item by providing additional information not in the ms, or briefly explain why the item is not applicable/relevant for your study

We have specified that "It is possible to use the program without any contact with therapists but, like in some previous trials [e.g., 27], trained psychology students kept minimal contact with the participants by email. The messages were short feedbacks on usage and motivation to use the program delivered every other week." (p. 7, Treatment)

5-xi) Report any prompts/reminders used

Report any prompts/reminders used: Clarify if there were prompts (letters, emails, phone calls, SMS) to use the application, what triggered them, frequency etc. It may be necessary to distinguish between the level of prompts/reminders required for the trial, and the level of prompts/reminders for a routine application outside of a RCT setting (discuss under item 21 – generalizability).

subitem not at all important

- 1 ○
- 2 ◉
- 3 ○
- 4 ○
- 5 ○

essential

Limpar seleção

Does your paper address subitem 5-xi? *

Copy and paste relevant sections from the manuscript (include quotes in quotation marks "like this" to indicate direct quotes from your manuscript), or elaborate on this item by providing additional information not in the ms, or briefly explain why the item is not applicable/relevant for your study

It is described that "Deprexis sends automatic daily messages with therapeutic contents to help in everyday life (e.g., becoming aware of unhelpful automatic thoughts, organizing the day, breaking down large problems into small steps, interacting with people, etc.)." And this also occurs also in the offered in the routine version of the program

5-xii) Describe any co-interventions (incl. training/support)

Describe any co-interventions (incl. training/support): Clearly state any interventions that are provided in addition to the targeted eHealth intervention, as ehealth intervention may not be designed as stand-alone intervention. This includes training sessions and support [1]. It may be necessary to distinguish between the level of training required for the trial, and the level of training for a routine application outside of a RCT setting (discuss under item 21 – generalizability.

subitem not at all important

1 (selected)

2

3

4

5

essential

Limpar seleção

Does your paper address subitem 5-xii? *

Copy and paste relevant sections from the manuscript (include quotes in quotation marks "like this" to indicate direct quotes from your manuscript), or elaborate on this item by providing additional information not in the ms, or briefly explain why the item is not applicable/relevant for your study

NA. There was no other intervention

6a) Completely defined pre-specified primary and secondary outcome measures, including how and when they were assessed

Does your paper address CONSORT subitem 6a? *

Copy and paste relevant sections from the manuscript (include quotes in quotation marks "like this" to indicate direct quotes from your manuscript), or elaborate on this item by providing additional information not in the ms, or briefly explain why the item is not applicable/relevant for your study

"Patient Health Questionnaire [35,36]. The PHQ-9 served as the primary outcome measure" (Instruments section), and it was consistent in other parts of the paper (eg, Sample Size section "The sample size calculation of this study was based on the expected difference in the primary outcome variable (PHQ-9, which measures the severity of depressive symptoms"; "we observe significantly higher main effects for the immediate access group for the primary outcome measure [PHQ-9; F (1, 173.5)=19.85, p < 0.001]"). Equally, the CORE-OM and GSES were explicitly the secondary measures (Instruments section; eg, in the Results, "for the secondary outcome measures, between-group effect sizes at post-treatment with estimated means (ITT samples) were medium to large, ranging from d=0.63 (self-efficacy measure) to d=0.82 (CORE-OM total score)"

6a-i) Online questionnaires: describe if they were validated for online use and apply CHERRIES items to describe how the questionnaires were designed/deployed

If outcomes were obtained through online questionnaires, describe if they were validated for online use and apply CHERRIES items to describe how the questionnaires were designed/deployed [9].

subitem not at all important

- 1 ○
- 2 ◉
- 3 ○
- 4 ○
- 5 ○

essential

Limpar seleção

Does your paper address subitem 6a-i?

Copy and paste relevant sections from manuscript text

We do not address subitem 6a-i using CHERRIES. What we did were calculating the internal consistency for all measures and we found that they were similar to other reports.

6a-ii) Describe whether and how “use” (including intensity of use/dosage) was defined/measured/monitored

Describe whether and how “use” (including intensity of use/dosage) was defined/measured/monitored (logins, logfile analysis, etc.). Use/adoption metrics are important process outcomes that should be reported in any ehealth trial.

subitem not at all important

1 ○

2 ○

3 ○

4 ◉

5 ○

essential

Limpar seleção

Does your paper address subitem 6a-ii?

Copy and paste relevant sections from manuscript text

We had limited access to this data, but managed to report the following "The participants from the immediate access group (n=44) used Deprexis on average 14.81 log-ins (SD=12.16)"

6a-iii) Describe whether, how, and when qualitative feedback from participants was obtained

Describe whether, how, and when qualitative feedback from participants was obtained (e.g., through emails, feedback forms, interviews, focus groups).

subitem not at all important

1 ○

2 ○

3 ○

4 ○

5 ◉

essential

Limpar seleção

Does your paper address subitem 6a-iii?

Copy and paste relevant sections from manuscript text

Since we had a high dropout rate, we asked the dropouts the reason for not answering the post-treatment questionnaires. "We examined the messages exchanged by the staff and 32 dropouts who answered the reasons for dropping out. We found that 16 participants reported that they had lost interest, 8 feared that the data would not be secure enough, two had problems with the login-in procedure and gave up trying, two reported that they did not present depressive symptoms anymore, two stated that questionnaires were too long, and one feared that he would be charged for it in the end."

6b) Any changes to trial outcomes after the trial commenced, with reasons

Does your paper address CONSORT subitem 6b? *

Copy and paste relevant sections from the manuscript (include quotes in quotation marks "like this" to indicate direct quotes from your manuscript), or elaborate on this item by providing additional information not in the ms, or briefly explain why the item is not applicable/relevant for your study

NA. The trial was conducted according to the trial registration https://ensaiosclinicos.gov.br/rg/RBR-6kk3bx/

7a) How sample size was determined

NPT: When applicable, details of whether and how the clustering by care provides or centers was addressed

7a-i) Describe whether and how expected attrition was taken into account when calculating the sample size

Describe whether and how expected attrition was taken into account when calculating the sample size.

subitem not at all important

1 ○

2 ○

3 ○

4 ○

5 ◉

essential

Limpar seleção

Does your paper address subitem 7a-i?

Copy and paste relevant sections from manuscript title (include quotes in quotation marks "like this" to indicate direct quotes from your manuscript), or elaborate on this item by providing additional information not in the ms, or briefly explain why the item is not applicable/relevant for your study

"Based on an estimate of statistical power of at least 0.80 in a two-tailed test and an alpha of 0.05, we calculated a sample size of 128 participants (64 per group) to be able to demonstrate a likely effect size of d=0.50 (medium effect, based on previous research with Deprexis; [medium effect, based on previous research with Deprexis; 16,52]. Based on previous clinical trials with Deprexis [e.g., 27], we anticipated a study dropout rate of 20%.". We also explain in the Results section why we collected more data than planned "Due to the high study dropout rate (i.e., participants not filling in the post-treatment assessments) and given that we had enough funding, we collected more data than the 128 initially planned [34]. We randomized 189 moderately and severely depressed participants"

7b) When applicable, explanation of any interim analyses and stopping guidelines

Does your paper address CONSORT subitem 7b? *

Copy and paste relevant sections from the manuscript (include quotes in quotation marks "like this" to indicate direct quotes from your manuscript), or elaborate on this item by providing additional information not in the ms, or briefly explain why the item is not applicable/relevant for your study

Again, we extended the data collection because of the high dropout rate and made that explicit in the Results section: "Due to the high study dropout rate (i.e., participants not filling in the post-treatment assessments) and given that we had enough funding, we collected more data than the 128 initially planned [34]. We randomized 189 moderately and severely depressed participants"

8a) Method used to generate the random allocation sequence

NPT: When applicable, how care providers were allocated to each trial group

Does your paper address CONSORT subitem 8a? *

Copy and paste relevant sections from the manuscript (include quotes in quotation marks "like this" to indicate direct quotes from your manuscript), or elaborate on this item by providing additional information not in the ms, or briefly explain why the item is not applicable/relevant for your study

"The selected participants were randomly assigned to either the immediate access condition or to the delayed access condition. A 50:50 randomization procedure was used. It was computer-generated using the random algorithm developed at http://www.randomization.com. The randomization scheme was concealed from the whole study team."

8b) Type of randomisation; details of any restriction (such as blocking and block size)

Does your paper address CONSORT subitem 8b? *

Copy and paste relevant sections from the manuscript (include quotes in quotation marks "like this" to indicate direct quotes from your manuscript), or elaborate on this item by providing additional information not in the ms, or briefly explain why the item is not applicable/relevant for your study

"A 50:50 randomization procedure was used"

9) Mechanism used to implement the random allocation sequence (such as sequentially numbered containers), describing any steps taken to conceal the sequence until interventions were assigned

Does your paper address CONSORT subitem 9? *

Copy and paste relevant sections from the manuscript (include quotes in quotation marks "like this" to indicate direct quotes from your manuscript), or elaborate on this item by providing additional information not in the ms, or briefly explain why the item is not applicable/relevant for your study

"It was computer-generated using the random algorithm developed at http://www.randomization.com. The randomization scheme was concealed from the study team."

10) Who generated the random allocation sequence, who enrolled participants, and who assigned participants to interventions

Does your paper address CONSORT subitem 10? *

Copy and paste relevant sections from the manuscript (include quotes in quotation marks "like this" to indicate direct quotes from your manuscript), or elaborate on this item by providing additional information not in the ms, or briefly explain why the item is not applicable/relevant for your study

It is now added that "The list was generated prior to the data collection by the study leader and only one student could open the file."

11a) If done, who was blinded after assignment to interventions (for example, participants, care providers, those assessing outcomes) and how

NPT: Whether or not administering co-interventions were blinded to group assignment

11a-i) Specify who was blinded, and who wasn't

Specify who was blinded, and who wasn't. Usually, in web-based trials it is not possible to blind the participants [1, 3] (this should be clearly acknowledged), but it may be possible to blind outcome assessors, those doing data analysis or those administering co-interventions (if any).

subitem not at all important

- 1 ○
- 2 ◉
- 3 ○
- 4 ○
- 5 ○

essential

Limpar seleção

Does your paper address subitem 11a-i? *

Copy and paste relevant sections from the manuscript (include quotes in quotation marks "like this" to indicate direct quotes from your manuscript), or elaborate on this item by providing additional information not in the ms, or briefly explain why the item is not applicable/relevant for your study

"Participants were not blinded to which condition they were randomized." Since we used a delayed intervention, participants were aware that they would only get the intervention later.

11a-ii) Discuss e.g., whether participants knew which intervention was the “intervention of interest” and which one was the “comparator”

Informed consent procedures (4a-ii) can create biases and certain expectations - discuss e.g., whether participants knew which intervention was the “intervention of interest” and which one was the “comparator”.

subitem not at all important

1 ○

2 ○

3 ◉

4 ○

5 ○

essential

Limpar seleção

Does your paper address subitem 11a-ii?

Copy and paste relevant sections from the manuscript (include quotes in quotation marks "like this" to indicate direct quotes from your manuscript), or elaborate on this item by providing additional information not in the ms, or briefly explain why the item is not applicable/relevant for your study

"Participants were randomly allocated into two conditions: (1) an experimental condition, in which they received treatment as usual (TAU) plus immediate access to Deprexis for 90 days or (2) a control group, in which they also received TAU and delayed access to Deprexis after 8 weeks. Participants were not blinded to which condition they were randomized. "

11b) If relevant, description of the similarity of interventions
(this item is usually not relevant for ehealth trials as it refers to similarity of a placebo or sham intervention to a active medication/intervention)

Does your paper address CONSORT subitem 11b? *

Copy and paste relevant sections from the manuscript (include quotes in quotation marks "like this" to indicate direct quotes from your manuscript), or elaborate on this item by providing additional information not in the ms, or briefly explain why the item is not applicable/relevant for your study

Not aplicable, only one intervention was used

12a) Statistical methods used to compare groups for primary and secondary outcomes
NPT: When applicable, details of whether and how the clustering by care providers or centers was addressed

Does your paper address CONSORT subitem 12a? *

Copy and paste relevant sections from the manuscript (include quotes in quotation marks "like this" to indicate direct quotes from your manuscript), or elaborate on this item by providing additional information not in the ms, or briefly explain why the item is not applicable/relevant for your study

"ITT analyses were conducted using linear mixed models, which are widely used in this field of research and have been recommended because of their ability to estimate the missing data accurately [54,55]. Analyses of those who adhere to the protocol (per Protocol, PP, or completer analysis) were conducted using a general linear model for repeated measures and taking into account the observed (actual) means and standard deviations. Pre-post-treatment effect sizes and between-groups effect sizes were evaluated using Cohen's d.The clinical significance of the changes observed at post-treatment and after the delayed period was evaluated using the Jacobson & Truax's method [56]. For participants with missing post-treatment or post-delayed period data, the Last Observation Carried Forward method [57] was used. Chi-square tests were used to compare the rates of clinical improvement, and recovery, as well as any clinically significant deterioration ratios between groups." And it was consistent with the study protocol (Lopes et al 2020)."

12a-i) Imputation techniques to deal with attrition / missing values

Imputation techniques to deal with attrition / missing values: Not all participants will use the intervention/comparator as intended and attrition is typically high in ehealth trials. Specify how participants who did not use the application or dropped out from the trial were treated in the statistical analysis (a complete case analysis is strongly discouraged, and simple imputation techniques such as LOCF may also be problematic [4]).

subitem not at all important

1 ○

2 ○

3 ○

4 ◉

5 ○

essential

Limpar seleção

Does your paper address subitem 12a-i? *

Copy and paste relevant sections from the manuscript (include quotes in quotation marks "like this" to indicate direct quotes from your manuscript), or elaborate on this item by providing additional information not in the ms, or briefly explain why the item is not applicable/relevant for your study

"ITT analyses were conducted using linear mixed models, which are widely used in this field of research and have been recommended because of their ability to estimate the missing data accurately [54,55]."
"The clinical significance of the changes observed at post-treatment and after the delayed period was evaluated using the Jacobson & Truax's method [56]. For participants with missing post-treatment or post-delayed period data, the Last Observation Carried Forward method [57] was used."

12b) Methods for additional analyses, such as subgroup analyses and adjusted analyses

Does your paper address CONSORT subitem 12b? *

Copy and paste relevant sections from the manuscript (include quotes in quotation marks "like this" to indicate direct quotes from your manuscript), or elaborate on this item by providing additional information not in the ms, or briefly explain why the item is not applicable/relevant for your study

"The clinical significance of the changes observed at post-treatment and after the delayed period was evaluated using the Jacobson & Truax's method [56]. For participants with missing post-treatment or post-delayed period data, the Last Observation Carried Forward method [57] was used. Chi-square tests were used to compare the rates of clinical improvement, and recovery, as well as any clinically significant deterioration ratios between groups."

X26) REB/IRB Approval and Ethical Considerations [recommended as subheading under "Methods"] (not a CONSORT item)

X26-i) Comment on ethics committee approval

subitem not at all important

1 ○

2 ○

3 ●

4 ○

5 ○

essential

Limpar seleção

Does your paper address subitem X26-i?

Copy and paste relevant sections from the manuscript (include quotes in quotation marks "like this" to indicate direct quotes from your manuscript), or elaborate on this item by providing additional information not in the ms, or briefly explain why the item is not applicable/relevant for your study

"This research protocol was approved by the Research Ethics Committee of the Catholic University of Petrópolis, which is recognized by the Brazilian Ministry of Health (CAAE # 68709517.1.0000.5281)."

x26-ii) Outline informed consent procedures

Outline informed consent procedures e.g., if consent was obtained offline or online (how? Checkbox, etc.?), and what information was provided (see 4a-ii). See [6] for some items to be included in informed consent documents.

subitem not at all important

1 ○

2 ○

3 ○

4 ◉

5 ○

essential

Limpar seleção

Does your paper address subitem X26-ii?

Copy and paste relevant sections from the manuscript (include quotes in quotation marks "like this" to indicate direct quotes from your manuscript), or elaborate on this item by providing additional information not in the ms, or briefly explain why the item is not applicable/relevant for your study

All selected clients who decide to be part of the research were informed and consented to participate by clicking “yes” on the online Informed Consent Form, in which they were explained the aim of the research, that Deprexis has been previously researched, that minimal risks were expected and they could withdraw at any time.

X26-iii) Safety and security procedures

Safety and security procedures, incl. privacy considerations, and any steps taken to reduce the likelihood or detection of harm (e.g., education and training, availability of a hotline)

subitem not at all important

1 ○

2 ○

3 ○

4 ◉

5 ○

essential

Limpar seleção

Does your paper address subitem X26-iii?

Copy and paste relevant sections from the manuscript (include quotes in quotation marks "like this" to indicate direct quotes from your manuscript), or elaborate on this item by providing additional information not in the ms, or briefly explain why the item is not applicable/relevant for your study

"The students kept an open channel so that the participants could report any eventual harming effects or if they had general questions about the program." (Methods/Intervention). We now added "No participant explicitly wrote the students about any harmful effects of the program" in the results section.

RESULTS

13a) For each group, the numbers of participants who were randomly assigned, received intended treatment, and were analysed for the primary outcome

NPT: The number of care providers or centers performing the intervention in each group and the number of patients treated by each care provider in each center

Does your paper address CONSORT subitem 13a? *

Copy and paste relevant sections from the manuscript (include quotes in quotation marks "like this" to indicate direct quotes from your manuscript), or elaborate on this item by providing additional information not in the ms, or briefly explain why the item is not applicable/relevant for your study

"As shown in the flowchart (Figure 2), many interested individuals clicked on the website (n=39.192). Out of 2.896 participants who completed the first screening, 2305 were invited for the interview and 312 were interviewed. Due to the high study dropout rate (i.e., participants not filling in the post-treatment assessments) and given that we had enough funding, we collected more data than the 128 initially planned [34]. We randomized 189 moderately and severely depressed participants to the immediate access condition (TAU plus immediate access to Deprexis for 90 days, n=94), or to the delayed access control group (TAU and delayed access to Deprexis, after 8 weeks, n= 95)."

13b) For each group, losses and exclusions after randomisation, together with reasons

Does your paper address CONSORT subitem 13b? (NOTE: Preferably, this is shown in a CONSORT flow diagram) *

Copy and paste relevant sections from the manuscript (include quotes in quotation marks "like this" to indicate direct quotes from your manuscript), or elaborate on this item by providing additional information not in the ms, or briefly explain why the item is not applicable/relevant for your study

The number and the reasons for losses are presented in detail in Fig 2 (study flowchart)

13b-i) Attrition diagram

Strongly recommended: An attrition diagram (e.g., proportion of participants still logging in or using the intervention/comparator in each group plotted over time, similar to a survival curve) or other figures or tables demonstrating usage/dose/engagement.

subitem not at all important

1 ○

2 ○

3 ○

4 ○

5 ◉

essential

Limpar seleção

Does your paper address subitem 13b-i?

Copy and paste relevant sections from the manuscript or cite the figure number if applicable (include quotes in quotation marks "like this" to indicate direct quotes from your manuscript), or elaborate on this item by providing additional information not in the ms, or briefly explain why the item is not applicable/relevant for your study

The number and the reasons for losses are presented in more detail in Fig 2 (study flowchart). A special subsection is addresses to dropout. "Dropout in this study is defined as not filling in the post-treatment questionnaires. We had 79 randomized participants dropping out of the study from pre- to post-treatment (out of 189; i.e., 41.79% in total, 53.19% of the immediate access group, and 30.52% of the delayed access control group). Of those 79 dropouts, 48 (25.39%)

14a) Dates defining the periods of recruitment and follow-up

Does your paper address CONSORT subitem 14a? *

Copy and paste relevant sections from the manuscript (include quotes in quotation marks "like this" to indicate direct quotes from your manuscript), or elaborate on this item by providing additional information not in the ms, or briefly explain why the item is not applicable/relevant for your study

"Recruitment of participants took place between August 2018 (first participant in) and September 2020 (last participant in)." No follow up data was collected until now.

14a-i) Indicate if critical "secular events" fell into the study period

Indicate if critical "secular events" fell into the study period, e.g., significant changes in Internet resources available or "changes in computer hardware or Internet delivery resources"

subitem not at all important

- 1 (selected)
- 2
- 3
- 4
- 5

essential

Limpar seleção

Does your paper address subitem 14a-i?

Copy and paste relevant sections from the manuscript (include quotes in quotation marks "like this" to indicate direct quotes from your manuscript), or elaborate on this item by providing additional information not in the ms, or briefly explain why the item is not applicable/relevant for your study

The data collection started before the pandemic. We made an analysis of the pre-treatment characteristics of participants joining before and after the pandemic (Table 1) and also tested whether it influenced dropout rates "intake before and after the beginning of the pandemic [x2(2)=1.86, P=.17]".

14b) Why the trial ended or was stopped (early)

Does your paper address CONSORT subitem 14b? *

Copy and paste relevant sections from the manuscript (include quotes in quotation marks "like this" to indicate direct quotes from your manuscript), or elaborate on this item by providing additional information not in the ms, or briefly explain why the item is not applicable/relevant for your study

NA. The trial was not ended or was stopped earlier than planned

15) A table showing baseline demographic and clinical characteristics for each group

NPT: When applicable, a description of care providers (case volume, qualification, expertise, etc.) and centers (volume) in each group

Does your paper address CONSORT subitem 15? *

Copy and paste relevant sections from the manuscript (include quotes in quotation marks "like this" to indicate direct quotes from your manuscript), or elaborate on this item by providing additional information not in the ms, or briefly explain why the item is not applicable/relevant for your study

Table 1 reports the demographical and clinical characteristics of both samples.

15-i) Report demographics associated with digital divide issues

In ehealth trials it is particularly important to report demographics associated with digital divide issues, such as age, education, gender, social-economic status, computer/Internet/ehealth literacy of the participants, if known.

subitem not at all important

1 ○

2 ○

3 ◉

4 ○

5 ○

essential

Limpar seleção

Does your paper address subitem 15-i? *

Copy and paste relevant sections from the manuscript (include quotes in quotation marks "like this" to indicate direct quotes from your manuscript), or elaborate on this item by providing additional information not in the ms, or briefly explain why the item is not applicable/relevant for your study

We report data regarding age, gender, recruitment source and clinical characteristics. We did not include a measure of ehealth literacy.

16) For each group, number of participants (denominator) included in each analysis and whether the analysis was by original assigned groups

16-i) Report multiple “denominators” and provide definitions

Report multiple “denominators” and provide definitions: Report N’s (and effect sizes) “across a range of study participation [and use] thresholds” [1], e.g., N exposed, N consented, N used more than x times, N used more than y weeks, N participants “used” the intervention/comparator at specific pre-defined time points of interest (in absolute and relative numbers per group). Always clearly define “use” of the intervention.

subitem not at all important

1 ○

2 ○

3 ○

4 ◉

5 ○

essential

Limpar seleção

Does your paper address subitem 16-i? *

Copy and paste relevant sections from the manuscript (include quotes in quotation marks "like this" to indicate direct quotes from your manuscript), or elaborate on this item by providing additional information not in the ms, or briefly explain why the item is not applicable/relevant for your study

Tables 2 and 3 are consistent in reporting the denominator n for each analysis

16-ii) Primary analysis should be intent-to-treat

Primary analysis should be intent-to-treat, secondary analyses could include comparing only “users”, with the appropriate caveats that this is no longer a randomized sample (see 18-i).

subitem not at all important

1 ○

2 ○

3 ○

4 ○

5 ◉

essential

Limpar seleção

Does your paper address subitem 16-ii?

Copy and paste relevant sections from the manuscript (include quotes in quotation marks "like this" to indicate direct quotes from your manuscript), or elaborate on this item by providing additional information not in the ms, or briefly explain why the item is not applicable/relevant for your study

This is consistent in the paper
Analysis section: "Following the intention-to-treat (ITT) principle, data from all randomized participants were analyzed. ITT analyses were conducted using linear mixed models, which are widely used in this field of research and have been recommended because of their ability to estimate the missing data accurately [54,55]. Analyses of those who adhere to the protocol (per Protocol, PP, or completer analysis) were conducted using a general linear model for repeated measures and taking into account the observed (actual) means and standard deviations. Pre-post-treatment effect sizes and between-groups effect sizes were evaluated using Cohen’s d.The clinical significance of the changes observed at post-treatment and after the delayed period was evaluated using the Jacobson & Truax’s method [56]. For participants with missing post-treatment or post-delayed period data, the Last Observation Carried Forward method [57] was used. Chi-square tests were used to compare the rates of clinical improvement, and recovery, as well as any clinically significant deterioration ratios between groups."
And throughout the results section (see tables 2 and 3).

17a) For each primary and secondary outcome, results for each group, and the estimated effect size and its precision (such as 95% confidence interval)

Does your paper address CONSORT subitem 17a? *

Copy and paste relevant sections from the manuscript (include quotes in quotation marks "like this" to indicate direct quotes from your manuscript), or elaborate on this item by providing additional information not in the ms, or briefly explain why the item is not applicable/relevant for your study

Table 2 shows the mean scores and standard deviations at pre-treatment, post-treatment, and post-delayed access period and the effect sizes, including CI.

17a-i) Presentation of process outcomes such as metrics of use and intensity of use

In addition to primary/secondary (clinical) outcomes, the presentation of process outcomes such as metrics of use and intensity of use (dose, exposure) and their operational definitions is critical. This does not only refer to metrics of attrition (13-b) (often a binary variable), but also to more continuous exposure metrics such as "average session length". These must be accompanied by a technical description how a metric like a "session" is defined (e.g., timeout after idle time) [1] (report under item 6a).

subitem not at all important

1 ○

2 ○

3 ◉

4 ○

5 ○

essential

Limpar seleção

Does your paper address subitem 17a-i?

Copy and paste relevant sections from the manuscript (include quotes in quotation marks "like this" to indicate direct quotes from your manuscript), or elaborate on this item by providing additional information not in the ms, or briefly explain why the item is not applicable/relevant for your study

It was not our goal to analyse usage, but still we present a that "The participants from the immediate access group (n=44) used Deprexis on average 14.81 days (SD=12.16)."

17b) For binary outcomes, presentation of both absolute and relative effect sizes is recommended

Does your paper address CONSORT subitem 17b? *

Copy and paste relevant sections from the manuscript (include quotes in quotation marks "like this" to indicate direct quotes from your manuscript), or elaborate on this item by providing additional information not in the ms, or briefly explain why the item is not applicable/relevant for your study

We present the absolute and relative numbers for the clinical analysis (see table 3)

18) Results of any other analyses performed, including subgroup analyses and adjusted analyses, distinguishing pre-specified from exploratory

Does your paper address CONSORT subitem 18? *

Copy and paste relevant sections from the manuscript (include quotes in quotation marks "like this" to indicate direct quotes from your manuscript), or elaborate on this item by providing additional information not in the ms, or briefly explain why the item is not applicable/relevant for your study

"Dropout in this study is defined as not filling in the post-treatment questionnaires. We had 79 randomized participants dropping out of the study from pre- to post-treatment (out of 189; i.e., 41.79% in total, 53.19% of the immediate access group, and 30.52% of the delayed access control group). Of those 79 dropouts, 48 (25.39%) dropped out after the interview and before commencing to use Deprexis (see Figure 1). We compared demographical and clinical characteristics of completers (n=110) and dropouts (n=79). We found no differences in regards to age [t (177) =-1.27, P=.21], gender [x2 (2)=0.61, P=.47], recruitment source [x2(2)=0.61, P=.47], intake before and after the beginning of the COVID-19 pandemic [x2(2)=1.86, P=.17], having a previous mental health diagnosis [x2(2)=1.13, P=.29], having comorbidities [x2(2)=0.28, P=.60], being in another mental health treatment [x2(2)=3.24, P=.07], baseline score of GSES [t (187)=0.24, P=.81)], baseline score of CORE-OM [t (170)=-.56, P=.58) or baseline score of PHQ-9 [t (187)=1.48, P=.14]. Dropouts used Deprexis significantly less (M=3.94 days of use) than completers [14.81 days of use; t(130)=-3.60, P<.001].
We examined the messages exchanged by the staff and 32 dropouts who answered the reasons for dropping out. We found that 16 participants reported that they had lost interest, 8 feared that the data would not be secure enough, two had problems with the login-in procedure and gave up trying, two reported that they did not present depressive symptoms anymore, two stated that questionnaires were too long, and one feared that he would be charged for it in the end."

18-i) Subgroup analysis of comparing only users

A subgroup analysis of comparing only users is not uncommon in ehealth trials, but if done, it must be stressed that this is a self-selected sample and no longer an unbiased sample from a randomized trial (see 16-iii).

subitem not at all important

1 ○

2 ○

3 ○

4 ◉

5 ○

essential

Limpar seleção

Does your paper address subitem 18-i?

Copy and paste relevant sections from the manuscript (include quotes in quotation marks "like this" to indicate direct quotes from your manuscript), or elaborate on this item by providing additional information not in the ms, or briefly explain why the item is not applicable/relevant for your study

We show the completer analysis at all analysis, including the clinical analysis (Table 3)

19) All important harms or unintended effects in each group

(for specific guidance see CONSORT for harms)

Does your paper address CONSORT subitem 19? *

Copy and paste relevant sections from the manuscript (include quotes in quotation marks "like this" to indicate direct quotes from your manuscript), or elaborate on this item by providing additional information not in the ms, or briefly explain why the item is not applicable/relevant for your study

No participant explicitly wrote the students about any harmful effects of the program.

19-i) Include privacy breaches, technical problems

Include privacy breaches, technical problems. This does not only include physical “harm” to participants, but also incidents such as perceived or real privacy breaches [1], technical problems, and other unexpected/unintended incidents. “Unintended effects” also includes unintended positive effects [2].

subitem not at all important

1 ○

2 ○

3 ◉

4 ○

5 ○

essential

Limpar seleção

Does your paper address subitem 19-i?

Copy and paste relevant sections from the manuscript (include quotes in quotation marks "like this" to indicate direct quotes from your manuscript), or elaborate on this item by providing additional information not in the ms, or briefly explain why the item is not applicable/relevant for your study

We did not experience any privacy breaches, technical problems

19-ii) Include qualitative feedback from participants or observations from staff/researchers

Include qualitative feedback from participants or observations from staff/researchers, if available, on strengths and shortcomings of the application, especially if they point to unintended/unexpected effects or uses. This includes (if available) reasons for why people did or did not use the application as intended by the developers.

subitem not at all important

1 (selected)

2

3

4

5

essential

Limpar seleção

Does your paper address subitem 19-ii?

Copy and paste relevant sections from the manuscript (include quotes in quotation marks "like this" to indicate direct quotes from your manuscript), or elaborate on this item by providing additional information not in the ms, or briefly explain why the item is not applicable/relevant for your study

We did not assess qualitatively the participants

DISCUSSION

22) Interpretation consistent with results, balancing benefits and harms, and considering other relevant evidence

NPT: In addition, take into account the choice of the comparator, lack of or partial blinding, and unequal expertise of care providers or centers in each group

22-i) Restate study questions and summarize the answers suggested by the data, starting with primary outcomes and process outcomes (use)

Restate study questions and summarize the answers suggested by the data, starting with primary outcomes and process outcomes (use).

subitem not at all important

1 ○

2 ○

3 ○

4 ○

5 ◉

essential

Limpar seleção

Does your paper address subitem 22-i? *

Copy and paste relevant sections from the manuscript (include quotes in quotation marks "like this" to indicate direct quotes from your manuscript), or elaborate on this item by providing additional information not in the ms, or briefly explain why the item is not applicable/relevant for your study

"This study addressed a gap in the literature on the effectiveness of internet-based interventions for depression in countries with lower literacy rates, such as Brazil, and in another linguistic and cultural background. Analyses with both the completer and the intention to treat samples showed that participants who received Deprexis improved significantly more than the delayed access control group on the main (depressive symptoms) and the secondary outcome measure (general psychological state), with large effect sizes. Also, participants in the active group improved significantly more on a perceived self-efficacy measure, which yielded a medium effect size. Participants reported medium to high levels of satisfaction with Deprexis. These results replicate previous findings by showing that Deprexis can facilitate symptomatic improvement over 3 months. It also extends previous research by demonstrating that this intervention is effective in a Brazilian sample, which differs culturally and linguistically from the previously studied populations Germany, Switzerland, and the US."

22-ii) Highlight unanswered new questions, suggest future research

Highlight unanswered new questions, suggest future research.

subitem not at all important

1 ○

2 ○

3 ○

4 ○

5 ◉

essential

Limpar seleção

Does your paper address subitem 22-ii?

Copy and paste relevant sections from the manuscript (include quotes in quotation marks "like this" to indicate direct quotes from your manuscript), or elaborate on this item by providing additional information not in the ms, or briefly explain why the item is not applicable/relevant for your study

"More studies should address the problem of dropout from internet interventions with creative designs. The challenges are to have clear definitions of dropping out and to contact the dropout participants afterward."
"Additional research is needed to examine whether improvements are maintained over longer periods of time and who is particularly likely to respond to or drop out from this form of treatment"

20) Trial limitations, addressing sources of potential bias, imprecision, and, if relevant, multiplicity of analyses

20-i) Typical limitations in ehealth trials

Typical limitations in ehealth trials: Participants in ehealth trials are rarely blinded. Ehealth trials often look at a multiplicity of outcomes, increasing risk for a Type I error. Discuss biases due to non-use of the intervention/usability issues, biases through informed consent procedures, unexpected events.

subitem not at all important

1 ○

2 ○

3 ○

4 ○

5 ◉

essential

Limpar seleção

Does your paper address subitem 20-i? *

Copy and paste relevant sections from the manuscript (include quotes in quotation marks "like this" to indicate direct quotes from your manuscript), or elaborate on this item by providing additional information not in the ms, or briefly explain why the item is not applicable/relevant for your study

It is addressed in the discussion issues with recruiting participants from the community over the internet

21) Generalisability (external validity, applicability) of the trial findings

NPT: External validity of the trial findings according to the intervention, comparators, patients, and care providers or centers involved in the trial

21-i) Generalizability to other populations

Generalizability to other populations: In particular, discuss generalizability to a general Internet population, outside of a RCT setting, and general patient population, including applicability of the study results for other organizations

subitem not at all important

1 ○

2 ○

3 ○

4 ○

5 ◉

essential

Limpar seleção

Does your paper address subitem 21-i?

Copy and paste relevant sections from the manuscript (include quotes in quotation marks "like this" to indicate direct quotes from your manuscript), or elaborate on this item by providing additional information not in the ms, or briefly explain why the item is not applicable/relevant for your study

"Deprexis has been studied for many years now and has been carefully translated and adapted to ten different languages, including the Brazilian Portuguese language and culture. These results show that this intervention is effective in different cultures, even with minimal adaptations. From a public health perspective, this is an important information to expand the reach of internet interventions for the ones who really needed, especially in countries with less access to mental health care. "

21-ii) Discuss if there were elements in the RCT that would be different in a routine application setting

Discuss if there were elements in the RCT that would be different in a routine application setting (e.g., prompts/reminders, more human involvement, training sessions or other co-interventions) and what impact the omission of these elements could have on use, adoption, or outcomes if the intervention is applied outside of a RCT setting.

subitem not at all important

1 (selected)

2

3

4

5

essential

Limpar seleção

Does your paper address subitem 21-ii?

Copy and paste relevant sections from the manuscript (include quotes in quotation marks "like this" to indicate direct quotes from your manuscript), or elaborate on this item by providing additional information not in the ms, or briefly explain why the item is not applicable/relevant for your study

"we consider a strength that almost half of the participants randomized to the delayed access control group were receiving other forms of treatment as usual (psychotherapy alone, pharmacotherapy alone, or both combined). This should be a more conservative control group, in contrast to the common practice of a typical waiting list control group in which participants are selected for not having any other form of treatment."

OTHER INFORMATION

23) Registration number and name of trial registry

Does your paper address CONSORT subitem 23? *

Copy and paste relevant sections from the manuscript (include quotes in quotation marks "like this" to indicate direct quotes from your manuscript), or elaborate on this item by providing additional information not in the ms, or briefly explain why the item is not applicable/relevant for your study

Trial registration: ReBec RBR-6kk3bx, UTN U1111-1212-8998

24) Where the full trial protocol can be accessed, if available

Does your paper address CONSORT subitem 24? *

Cite a Multimedia Appendix, other reference, or copy and paste relevant sections from the manuscript (include quotes in quotation marks "like this" to indicate direct quotes from your manuscript), or elaborate on this item by providing additional information not in the ms, or briefly explain why the item is not applicable/relevant for your study

We used a randomized controlled trial (RCT) parallel-group design (trial registration at ReBec: RBR-6kk3bx, UTN U1111-1212-8998, https://ensaiosclinicos.gov.br/rg/RBR-6kk3bx/) [34].

25) Sources of funding and other support (such as supply of drugs), role of funders

Does your paper address CONSORT subitem 25? *

Copy and paste relevant sections from the manuscript (include quotes in quotation marks "like this" to indicate direct quotes from your manuscript), or elaborate on this item by providing additional information not in the ms, or briefly explain why the item is not applicable/relevant for your study

The study received financial support from Conselho Nacional de Desenvolvimento Científico e Tecnológico (CNPq), Fundação Carlos Chagas Filho de Amparo à Pesquisa do Estado do Rio de Janeiro (FAPERJ [IC/2017_1]), Fundação Cultural Dom Manoel Pedro da Cunha Cintra (FDC) and the Swiss National Science Foundation (through a Scientific Exchange Grant [IZSEZ0_189796] to R. Lopes and the OPTIMIZE project funding to T. Berger [10001C_197475]).

X27) Conflicts of Interest (not a CONSORT item)

X27-i) State the relation of the study team towards the system being evaluated

In addition to the usual declaration of interests (financial or otherwise), also state the relation of the study team towards the system being evaluated, i.e., state if the authors/evaluators are distinct from or identical with the developers/sponsors of the intervention.

subitem not at all important

1 ○

2 ○

3 ○

4 ◉

5 ○

essential

Limpar seleção

Does your paper address subitem X27-i?

Copy and paste relevant sections from the manuscript (include quotes in quotation marks "like this" to indicate direct quotes from your manuscript), or elaborate on this item by providing additional information not in the ms, or briefly explain why the item is not applicable/relevant for your study

The authors declare that they have no competing interests, with the following exception: B. Meyer is employed full-time as the research director at Gaia AG, the company that developed and owns the Deprexis program. B. Meyer has not been involved in the data collection, data organization, or analysis.

About the CONSORT EHEALTH checklist

As a result of using this checklist, did you make changes in your manuscript? *

- ( ) yes, major changes
- (x) yes, minor changes
- ( ) no

What were the most important changes you made as a result of using this checklist?

The abstract was expanded and information about possible harmful effects

How much time did you spend on going through the checklist INCLUDING making changes in your manuscript *

Around 6 hours (including one hour that I needed to re-do because the form became empty)

As a result of using this checklist, do you think your manuscript has improved? *

- (x) yes
- ( ) no
- ( ) Outro:

Would you like to become involved in the CONSORT EHEALTH group?

This would involve for example becoming involved in participating in a workshop and writing an "Explanation and Elaboration" document

- ○ yes
- ◉ no
- ○ Outro:

Limpar seleção

Any other comments or questions on CONSORT EHEALTH

Thanks!

Your answer must have a minimum of 25 characters.

STOP - Save this form as PDF before you click submit

To generate a record that you filled in this form, we recommend to generate a PDF of this page (on a Mac, simply select "print" and then select "print as PDF") before you submit it.

When you submit your (revised) paper to JMIR, please upload the PDF as supplementary file.

Don't worry if some text in the textboxes is cut off, as we still have the complete information in our database. Thank you!

Final step: Click submit !

Click submit so we have your answers in our database!

Enviar Limpar formulário

Nunca envie senhas pelo Formulários Google.

Este conteúdo não foi criado nem aprovado pelo Google. Denunciar abuso - Termos de Serviço - Política de Privacidade

Google Formulários
